# Characterization of β‑Carboline Derivatives Reveals a High Barrier to Resistance and Potent Activity against Ring-Stage and DHA-Induced Dormant *Plasmodium falciparum*

**DOI:** 10.1021/acsinfecdis.5c00714

**Published:** 2025-10-17

**Authors:** Reagan S. Haney, Joshua H. Butler, Lyric A. Wardlaw, Emilio F. Merino, Victoria Mendiola, Caitlin A. Cooper, Jopaul Mathew, Patrick K. Tumwebaze, Philip J. Rosenthal, Roland A. Cooper, Dennis E. Kyle, Zaira Rizopoulos, Delphine Baud, Stephen Brand, Maxim Totrov, Paul R. Carlier, Maria Belen Cassera

**Affiliations:** † Department of Biochemistry and Molecular Biology, 1355 University of Georgia, Athens, Georgia 30602, United States; ‡ Center for Tropical and Emerging Global Diseases, University of Georgia, Athens, Georgia 30602, United States; § Department of Chemistry, Virginia Tech, Blacksburg, Virginia 24061, United States; ∥ 560866 Infectious Diseases Research Collaboration, Kampala, Uganda; ⊥ Department of Medicine, University of California, San Francisco, California 94110, United States; # Department of Natural Sciences and Mathematics, 7178 Dominican University of California, San Rafael, California 94901, United States; ∇ Department of Cellular Biology, University of Georgia, Athens, Georgia 30602, United States; ○ Department of Infectious Diseases, University of Georgia, Athens, Georgia 30602, United States; ◆ 127356 Medicines for Malaria Venture, Geneva 1215, Switzerland; ¶ MolSoft LLC, San Diego, California 92121, United States; ⋈ Department of Pharmaceutical Sciences, 14681 University of Illinois Chicago, Chicago, Illinois 60612, United States

**Keywords:** malaria, β-carbolines, collateral drug sensitivity, dormancy, kelch-13, irresistible

## Abstract

Malaria, caused by *Plasmodium falciparum*, remains a major global health challenge, with an estimated 263 million new infections and 597,000 deaths annually. Increasing resistance to current antimalarial drugs underscores the urgent need for new therapeutics that target novel pathways in the parasite. We previously reported a novel class of β-carboline antimalarials, exemplified by PRC1584, which demonstrated a favorable oral pharmacokinetic profile, *in vivo* efficacy in *Plasmodium berghei*-infected mice, and no cross-resistance with other antimalarials in various *P. falciparum* strains. In this study, we demonstrate that PRC1584 exhibits a high resistance barrier and retains potent activity against fresh Ugandan *P. falciparum* isolates. PRC1584, along with its more potent analog PRC1697, demonstrated strong *in vitro* potency against both actively proliferating ring stages and dihydroartemisinin-induced dormant stages. Additionally, our study demonstrated that PfKelch13-C580Y mutation was associated with an increased susceptibility to PRC1584, whereas PfKelch13-R549T and Pfcoronin-R100 K-E107V mutations were not associated with this effect. These findings underscore the therapeutic potential of this new “irresistible” compound class, support a possible novel mechanism of action, and suggest the future development of novel ACTs active against resistant parasites by targeting DHA dormancy, an essential survival mechanism of *P. falciparum*.

## Graphical abstract

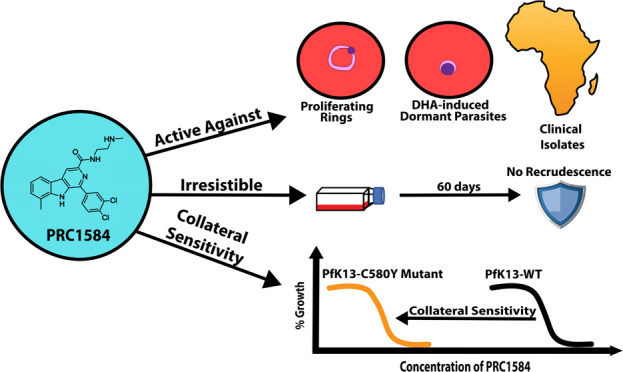

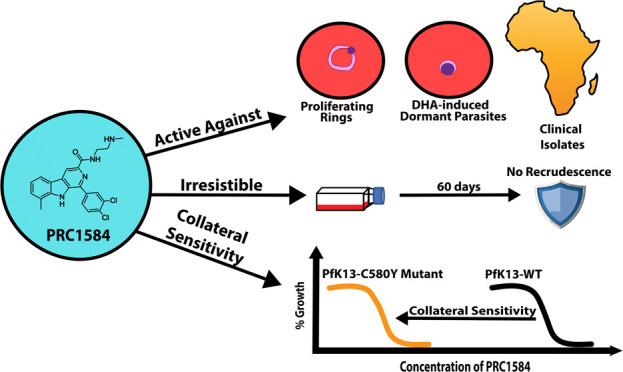

Malaria is a deadly disease caused by parasites of the genus *Plasmodium*, with *Plasmodium falciparum* being the deadliest of the human-infecting species. In 2023, an estimated 263 million malaria cases and 597,000 deaths were reported worldwide.^1^ Despite considerable progress over the last two decades in the fight against malaria, the current trend indicates a standstill. Chemotherapy remains a cornerstone of malaria control alongside vector management, new vaccines, diagnosis, and prompt access to treatment.^1^ However, declining clinical efficacy and rising resistance affect all currently used antimalarial drug classes, including artemisinin-based combination therapies (ACTs),^2^ which pair fast-acting artemisinin derivatives with slower-acting antimalarial drugs and remain the first-line treatment for malaria.^3^

Artemisinin partial resistance (ART-R) first emerged in the Greater Mekong subregion of Southeast Asia in 2008 ^4^ and has since also emerged in Africa.^5^ Clinically, ART-R is defined by a delay in parasite clearance following the initiation of an ACT regimen,^1^ and it is associated with reduced susceptibility in the early ring stages of the *P. falciparum* intraerythrocytic life cycle. This phenotype is assessed *in vitro* by the ring-stage survival assay (RSA_0–3 h_), which measures survival of early ring-stage parasites (0–3 h postinvasion) at 72 h postincubation with 700 nM dihydroartemisinin (DHA) for 6 h. This assay identifies resistant parasites with a survival rate over 1%, compared to untreated controls.^6^ Resistance to artemisinin is linked to single nucleotide polymorphisms (SNPs) in *pfkelch13* gene, which encodes the Kelch13 protein (K13).^3^ Although the exact function of PfKelch13 remains unclear, it is known that it is involved in hemoglobin uptake and endocytosis,^7^ regulation of phosphatidylinositol-3-phosphate vesicles enriched with proteins involved in proteostasis,^8^ unfolded protein response (UPR), and redox stress mitigation.^9^ Notably, many SNPs have been reported but PfKelch13-C580Y and R539T mutations are among the validated mutations strongly linked to ART-R,^3^ displaying elevated survival rates in the RSA_0–3 h_ assay.^3,10^

One survival mechanism in DHA-exposed parasites, regardless of their genetic background, is the temporary arrest of growth during the ring stage, known as dormancy.^11,12^ This is a phenomenon in which subpopulations of parasites, termed persisters, shutdown energy-related processes, and cease growth and proliferation.^9^ Phenotypically, dormancy is characterized as small (pyknotic) parasites with condensed nuclei and a reduced cytoplasm. This dormancy allows parasites to tolerate treatment, increasing the risk of clinical failure as they can later resume proliferation, a process known as recrudescence.^13^ A recent study showed that DHA-induced dormant parasites exhibit characteristics of cellular quiescence and senescence, meaning they temporarily halt growth and metabolic activity while maintaining viability.^14^ This work also showed dormancy requires a five-day maturation process during which gene expression gradually shifts from a ring-like state to a unique pattern different from that observed in the intraerythrocytic asexual and sexual stages. This slow transcriptional shift contrasts with the quick morphological change to pyknotic forms that occurs within 24 h, with parasites acquiring an irregular cellular ultrastructure, indicative of distinct biological characteristics. Therefore, the ability of malaria parasites to enter dormancy adds complexity to the challenge of developing novel antimalarials targeting the ring stage to reduce the pathogenesis and block transmission.

During ACT treatment, the artemisinin component has a short duration of efficacy resulting in rapid parasite clearance; the partner drug, which acts more slowly, has much longer duration of pharmacologically active concentrations.^15^ With the spread of ART-R, having an effective partner drug that eliminates dormant parasites or remains in the bloodstream long enough to kill parasites as they exit their DHA-induced dormant state to prevent recrudescence is crucial for the success of new ACTs.^16^

We recently identified a new class of β-carboline antimalarials, exemplified by PRC1584, which demonstrated a favorable oral pharmacokinetic profile, *in vivo* efficacy in *Plasmodium berghei*-infected mice, and low cross-resistance with other antimalarials in both susceptible and drug-resistant *P. falciparum* strains.^17^ Structure–activity relationship studies^18^ revealed a preference for a 3,4-dichloro- or 3,4,5-trichlorophenyl ring, with the most potent analog, PRC1697 (^Dd2^EC_50_ = 54 ± 8 nM), exhibiting twice the potency of PRC1584 (^Dd2^EC_50_ = 108 ± 7 nM). Barcoded cross-resistance profiling confirmed no cross-resistance against 32 resistance mutations in the Dd2 background and 10 in the 3D7 background, strongly suggesting that compounds within this scaffold possess a novel mechanism of antimalarial action.^18^

In this report, we describe a comprehensive evaluation of PRC1584 across key points of the parasite life cycle and its inability to select resistant parasites *in vitro*. Importantly, we demonstrate that PRC1584 is effective against both ring stages and DHA-induced dormant forms of *P. falciparum* parasites, regardless of their sensitivity or resistance to DHA. Together, these findings underscore the therapeutic potential of this “irresistible” β-carboline class of antimalarials that may act through a novel mechanism of action. The distinctive properties of this class provide opportunities for the advancement of innovative new combinations, including potentially future ACTs, particularly in the setting of ART-R, by specifically targeting DHA-induced dormancy, a critical survival strategy of *P. falciparum*.

## Results

### Activity of PRC1584 across the *P. falciparum* Life Cycle

In our first study, we identified PRC1584 (^Dd2^EC_50_ = 108 ± 7 nM) as the most potent analog among the new class of β-carbolines against asexual intraerythrocytic stages of *P. falciparum* (Figure 1A).^17^ Based on the promising activity of this early lead compound, we next tested PRC1584 against transmission and liver stages.

**Figure 1:**
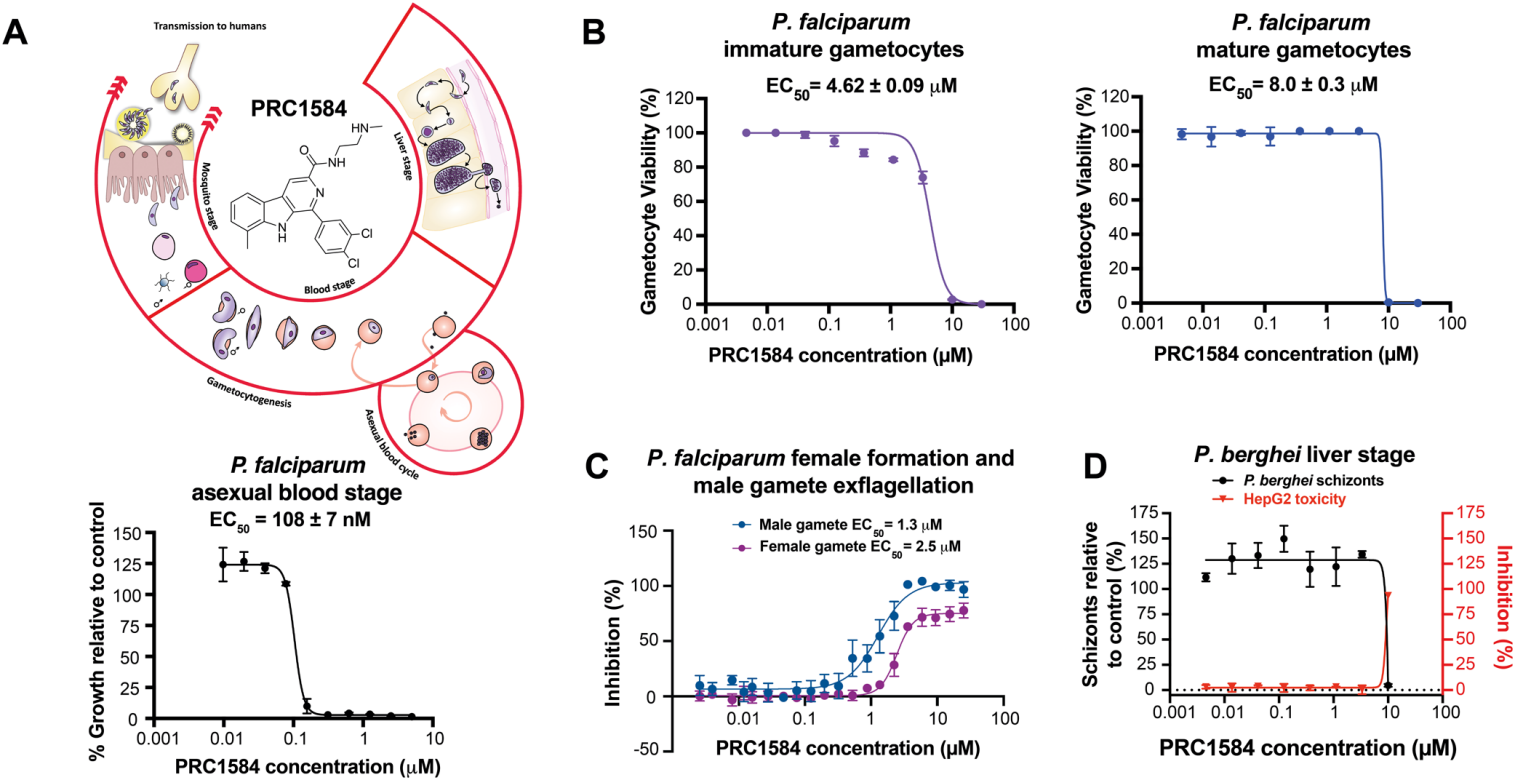
Antiplasmodial activity of PRC1584 throughout the *Plasmodium* parasite life cycle. (A) Schematic representation of the *P. falciparum* life cycle and chemical structure of PRC1584. Dose-dependent activity of PRC1584 against the *P. falciparum* Dd2 strain. (B) Dose-dependent activity of PRC1584 against immature (stage II/III) and mature (stage V) *P. falciparum* gametocytes following 48 h of exposure. Data represent the mean ± SEM from three independent biological replicates, each performed in technical triplicate. (C) Assessment of the ability of *P. falciparum* stage V gametocytes to undergo gametogenesis after 48 h of dose-dependent exposure to PRC1584. Data represent mean from one biological replicate performed in technical triplicate. (D) Dose-dependent activity of PRC1584 on *P. berghei* schizont development and HepG2 cell viability. Data represent the mean from two biological replicates performed in technical duplicates. All *x*-axes are shown on a logarithmic scale.

Potential transmission-blocking activity was assessed by measuring the stage-specific action of PRC1584 against immature and mature *P. falciparum* gametocytes (MMV, University of Pretoria). Gametocytes were induced from the PfNF54-*pfs16*-GFP-luc reporter line. Drug assays began on day 5 with gametocytes at stage II/III (immature) and on day 13 with gametocytes at stage V (mature). In each case, parasites were exposed to PRC1584 at varying concentrations for 48 h. Micromolar activity was observed against immature (EC_50_ = 4.62 ± 0.09 μM) and mature gametocytes (EC_50_ = 8.0 ± 0.3 μM) (Figure 1B).

To further assess the potential for β-carbolines to block transmission, the viability of mature stage V gametocytes was evaluated by their ability to develop into male and female gametes (MMV, Imperial College London). Since mature stage V gametocytes exhibit sex-specific sensitivity to antimalarials, both sexes should be studied to better characterize compound transmission-blocking activity.^19^ Therefore, compounds were incubated with mature stage V gametocytes for 48 h before inducing gamete formation by reducing the temperature and adding xanthurenic acid. Approximately 25 min postinduction, male gamete exflagellation was recorded and quantified using automated microscopy. Samples were then incubated at 26 °C for an additional 24 h, after which female gamete formation was assessed by live staining using a fluorophore-conjugated αPfs25 antibody specific for female gametes and quantified by automated microscopy. PRC1584 exhibited similar potency against male (EC_50_ = 1.3 μM) and female gametes (EC_50_ = 2.5 μM, Figure 1C).

Finally, we assessed the potential of PRC1584 in inhibiting liver-stage development of *P. berghei*. No inhibition was seen at concentrations up to 5 μM, with activity but also toxicity to HepG2 cells observed at 10 μM (Figure 1D). Collectively, these results demonstrate that PRC1584 displays significant *in vitro* potency primarily during the asexual intraerythrocytic cycle while exhibiting limited activity against gametocytes and liver-stage parasites.

### PRC1584 Retains Potency against Field Isolates

We previously established that PRC1584 is not subject to known resistance mechanisms of several antimalarials, including chloroquine, mefloquine, quinine, pyrimethamine, cycloguanil, sulfadoxine, KAE609, and dihydroartemisinin.^17^ This lack of cross-reactivity was further confirmed using the Antimalarial Resistome Barcode Sequencing (AReBar) pooled-screening assay.^18^ To determine whether resistance to PRC1584 is already present in parasites circulating in Africa, *ex vivo* sensitivity to PRC1584 was evaluated in fresh clinical isolates of *P. falciparum* obtained in Tororo, Uganda. At the time of these assays, isolates in Tororo were generally susceptible to most standard antimalarial drugs (chloroquine, monodesethylamodiaquine, piperaquine, pyronaridine, lumefantrine, mefloquine, and DHA) but resistant to pyrimethamine.^20^ The mean EC_50_ value for PRC1594 was found to be 70 nM, with a range of 26–177 nM (*n* = 31), which is consistent with potencies observed in laboratory strains (Figure 2 and Table S1). The mean EC_50_ for DHA in these isolates was 1.8 nM, with a range of 0.5–4.4 nM, while for chloroquine it was 17.2 nM with a range of 6.9–27 nM. Although these *ex vivo* results exhibited a 7-fold variation, which may be attributed to the complexity involved in assessing polyclonal isolates, all values remained within the low nanomolar range, suggesting that field isolates are consistently highly sensitive to PRC1584.

**Figure 2:**
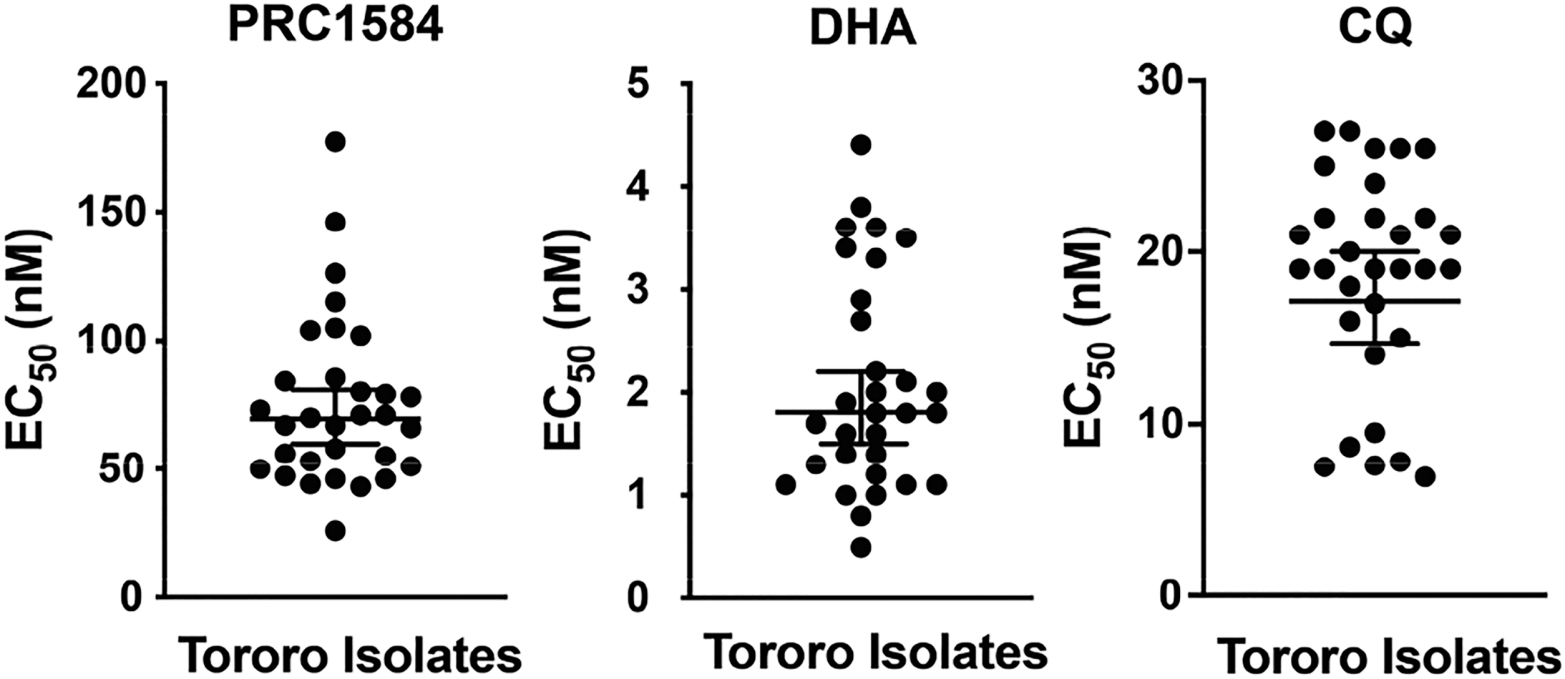
*Ex vivo* sensitivity to PRC1584 in fresh clinical isolates of *P. falciparum* obtained from Uganda (*n* = 31). Dose–response curves and EC_50_ values for PRC1584 (70 nM), DHA (1.8 nM), and chloroquine (CQ, 17.2 nM) were determined using a 72 h SYBR Green I growth inhibition assay. Data points represent individual isolates; horizontal bars indicate mean EC_50_ values, and error bars represent the standard error of the mean (SEM).

### PRC1584 is Refractory to Resistance Selection *In Vitro*

To gain further insight into the molecular targets or pathways that drive the significant antiplasmodial activity of PRC1584 against the asexual intraerythrocytic stages, we carried out multiple resistance selection protocols with the drug-sensitive *P. falciparum* 3D7 strain (Figure S1).^21–23^

Initially, a single step of resistance selection was performed by subjecting flasks containing an inoculum of 1 × 10^9^ parasites to PRC1584 at a concentration of five times the EC_50_ value (^3D7‑clone^EC_50_ = 67 ± 5 nM; Figure S2) for 14 days. In parallel, a flask treated with an equal volume of DMSO was used as the reference control. After the drug pressure was removed, cultures were monitored for 60 days. A second single step of resistance selection was performed using an inoculum of 1 × 10^7^ parasites and a concentration of 100 nM for 14 days (Figure S2). After removing drug pressure, cultures were monitored for 60 days. In both conditions, parasite clearance occurred within the first 4 days, and no parasite recrudescence was observed. From these experiments, the minimum inoculum of resistance (MIR) was determined to be >9 (i.e., >10^9^ infected red blood cells).

These findings were further validated using the *P. falciparum* Dd2-B2 clone (EC_50_ = 69 and EC_90_ = 136 nM). Parasites were subjected to constant drug pressure for 60 days at three times the EC_90_ (408 nM). Parasite clearance occurred within the first 4 days, and no recrudescence was observed over 60 days. In contrast, with the control DSM265 tested in parallel at five times the EC_50_ value of 57 nM, 68/96 wells yielded recrudescent parasites (Log_10_ MIR = 5.45).

We also attempted resistance selection by pulsing parasites for 6 h with eight times the 72 h EC_50_ value of PRC1584 (400 nM), followed by drug removal to allow parasites to recover before repeating the pulse (Figure S1B).^23^ This approach involves exposing parasites to elevated concentrations of the drug for a short period of time, resulting in the elimination of most parasites, followed by a drug-free period to enable the population recovery. The process of alternating drug exposure is continued until the parasites no longer responded to the treatment. The pulse-exposure method was performed with both highly synchronous rings (12–16 h postinvasion) and asynchronous cultures. Parasites reemerged during each of the four drug pulse cycles over a 30-day period, but all cultures died after the fourth cycle, preventing further *in vitro* assessments of PRC1584 potency. Across all conditions tested, including prolonged drug pressure and repeated pulsed exposure, no parasites were recovered. These findings provide evidence that PRC1584 exhibits a high barrier for resistance.

### PRC1584 Is Most Active against Late Ring and Early Trophozoite Stages

Our assessment across the *P. falciparum* life cycle revealed that PRC1584 exhibits significant activity primarily during the asexual intraerythrocytic cycle. To determine if PRC1584 targets specific stages within this cycle, we performed the asexual blood-stage-specificity inhibition assay described by Murithi and colleagues.^24^ This assay also enables the identification of potential mechanisms of action for new antimalarial drugs by comparing their susceptibility profiles to those of established antimalarials. As shown in Figure 3, PRC1584 displayed enhanced potency against parasites in the late ring and early trophozoite stages. Strikingly, an 8 h exposure during the early trophozoite stage yielded an ^3D7^EC_50_^8 h^ value of 192 ± 52 nM, slightly greater than the EC_50_ value observed with continuous 72 h exposure (^3D7^EC_50_^72 h^ = 108 ± 7 nM) (Figure 3C). Microscopy assessments of Giemsa-stained thin blood smears confirmed this stage-specific profile (Figure 3A). Interestingly, despite the biphasic dose-dependent curve shown in the 24–32 h window (Figure 3B), when parasites transition from late trophozoite stages to schizont stages, PRC1584-treated parasites remained arrested in the late trophozoite stage (Figure 3A). Moreover, the stage-specific dose–response profile of PRC1584 did not match the stage-specificity profile of any of the 36 clinical or experimental antimalarials reported by Murithi and colleagues,^24^ suggesting that this compound class may act through a distinct and potentially novel mechanism.

**Figure 3:**
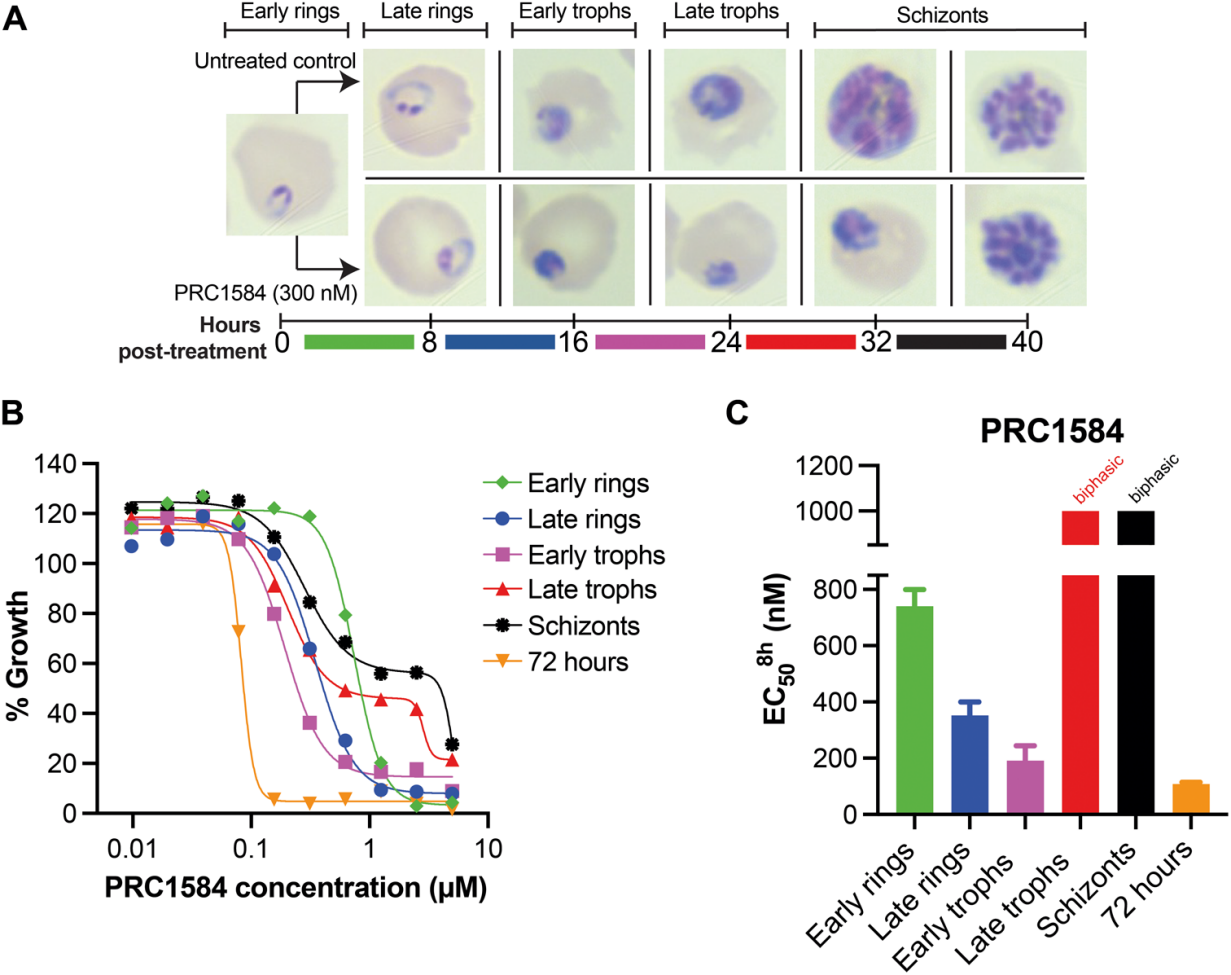
Stage-specificity inhibition profile for PRC1584. (A) Schematic of the intraerythrocytic life cycle of *P. falciparum* used for drug susceptibility assessment. Timeline represents 8 h treatment windows. Giemsa-stained images were captured at the treatment initiation (0 h) and after an 8 h drug exposure at the time of treatment removal, comparing untreated controls (top row) with parasites exposed to 300 nM PRC1584 (bottom row). (B) Dose–response curves of *P. falciparum* (3D7 strain) exposed for 8 h at defined development stages (early ring, late ring, early trophozoite (trophs), late trophozoites, and schizont). A 72 h continuous exposure curve was performed in parallel as a control. The *x*-axis for the growth inhibition curve is displayed on a logarithmic scale. (C) Bar plots showing the EC_50_^8 h^ values when parasites were exposed only during the indicated stage, as shown in panel A, with error bars displaying the SEM based on three biological replicates conducted in technical duplicate.

### The Kelch 13-C580Y Mutation Increases Susceptibility to PRC1584 and Its Analogs

We previously reported that PRC1584 remained equipotent against the *P. falciparum* 4G strain, which has reduced susceptibility to artemisinin, using a 72 h continuous exposure assay.^17^ However, previous studies have established that ART-R is associated with reduced susceptibility specifically in the ring stage.^25,26^ To better evaluate the susceptibility of the ring stage to PRC1584, we performed the stage-specificity assay described above, comparing the *P. falciparum* Dd2 (DHA-susceptible) strain with the 4G strain carrying the PfKelch13-C580Y mutation, which confers ART-R.^27^ Strikingly, PRC1584 was significantly more potent against the 4G strain than the Dd2 strain (^Dd2‑PRC1584^EC_50_^8 h^ = 696 ± 62 nM vs ^4G‑PRC1584^EC_50_^8 h^ = 211 ± 55 nM; Figures 4A and S3A). As expected, DHA was significantly less potent against the 4G strain compared to the Dd2 strain (^Dd2‑DHA^EC_50_^8 h^ = 3.0 ± 0.2 nM vs ^4G‑DHA^EC_50_^8 h^ = 7.7 ± 1.1 nM, Figure 4A). This unexpected collateral drug sensitivity was confirmed by comparing the susceptibility of PRC1584 in the NF54 wildtype strain and NF54-K13-C580Y line, expressing K13 wildtype (WT) or C580Y mutation, respectively^28^ (^NF54‑WT‑PRC1584^EC_50_^8 h^ = 476 ± 16 nM vs ^NF54‑K13‑C580Y‑PRC1584^EC_50_^8 h^ = 200 ± 56 nM; Figures 4B and S3B). The EC_50_ value for DHA was slightly but not significantly decreased in the NF54-K13-C580Y line compared to NF54-WT (^NF54‑WT‑DHA^EC_50_^8 h^ = 10 ± 2 nM vs ^NF54‑K13‑C580Y‑DHA^EC_50_^8 h^ = 6 ± 2 nM; Figure 4B). In addition, the potency of PRC1584 after 8 h exposure was slightly higher against the NF54-WT strain compared to Dd2 (^NF54‑WT‑PRC1584^EC_50_^8 h^ = 476 ± 16 nM vs ^Dd2‑PRC1584^EC_50_^8 h^ = 696 ± 62 nM), likely due to differences in the genetic background. Importantly, PRC1584 maintained strong potency against both 4G and NF54-K13-C580Y strains after an 8 h exposure (Figure 4A,B). Time-dependent effects were also observed, as 6 h exposure to PRC1584 led to increased EC_50_ values against both the WT strain and PfK13-C580Y line. Nevertheless, the PfK13-C580Y mutant continued to exhibit greater sensitivity to PRC1584 (^NF54‑WT‑PRC1584^EC_50_^6 h^ = 987 ± 64 nM vs ^NF54‑K13‑C580Y‑PRC1584^EC_50_^6 h^ = 394 ± 51 nM; Figure S4 and Table S1).

**Figure 4:**
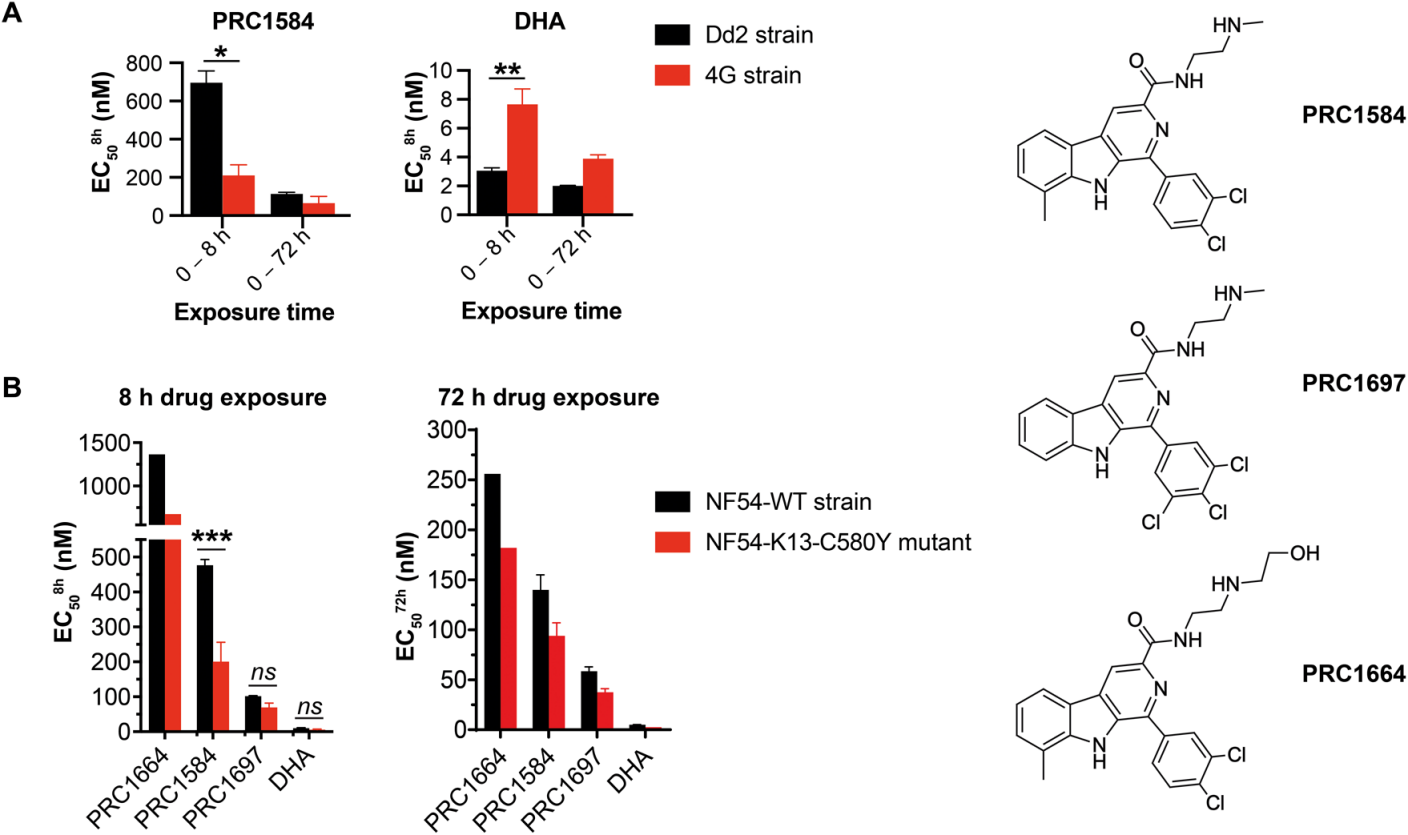
Collateral drug sensitivity in the presence of the PfK13-C580Y mutation was assessed by the stage-specificity profiling assay. (A) Early ring-stage parasites for both susceptible (Dd2) and resistant (4G) strains were exposed to PRC1584 and DHA in a dose-dependent manner for 8 h. A dose-dependent curve for 72 h continuous exposure was used as a control. Data represent the mean and SEM based on two to three independent biological replicates performed in technical triplicates. (B) Similar stage-specificity assay, as described in panel (A), was performed with NF54-WT strain and NF54-K13-C580Y mutant. Data represent one or two biological replicates performed in technical duplicates. Statistical comparisons were performed using the two-sample student’s *t* test. *p < 10^–5^; **p < 2 × 10^–5^; ***p < 10^–4^; *ns*, not significant. Sensitivity to PRC1697 increased, but the change was not statistically significant. Dose–response curves are shown in Figure S3.

To test whether this effect extended to other K13 mutations, we assessed PRC1584 against the Dd2-K13- R549T mutant, another DHA-resistant line. No differences were observed between Dd2-WT and Dd2-K13-R549T after 6 h of exposure (^Dd2‑WT‑PRC1584^EC_50_^6 h^ = 1630 ± 170 nM vs ^Dd2‑K13‑R549T‑PRC1584^EC_50_^6 h^ = 1950 ± 610 nM; Figure S5 and Table S1). We also evaluated PRC1584 against parasites carrying Pfcoronin mutations (Pikine-Pfcoronin-R100 K-E107V), which also appear to mediate resistance to DHA.^29^ The Pikine-Pfcoronin-R100 K-E107V mutant line exhibited similar susceptibility to PRC1584 as the parental Pikine strain after 6 and 8 h of exposure (^Pikine‑WT‑PRC1584^EC_50_^6 h^ = 530 nM vs ^Pikine‑Pfcoronin‑R100 K‑E107V‑PRC1584^EC_50_^6 h^ = 534 nM; ^Pikine‑WT‑PRC1584^EC_50_^8 h^ = 753 nM vs ^Pikine‑Pfcoronin‑R100 K‑E107V‑PRC1584^EC_50_^8 h^ = 604 nM; Figure S6). These data suggest that the PfK13-C580Y mutation specifically contributes to the increased potency for PRC1584.

We further examined whether collateral drug sensitivity extends to other β-carboline analogs recently reported in this series, specifically PRC1697 and PRC1664 (Figure 4B).^18^ Both analogs showed a similar profile of increased potency in the NF54-K13-C580Y mutant compared to NF54-WT (^NF54‑WT‑PRC1697^EC_50_^8 h^ = 102 ± 2 nM vs ^NF54‑K13‑C580Y‑PRC1697^EC_50_^8 h^ = 70 ± 13 nM; ^NF54‑WT‑PRC1664^EC_50_^8 h^ = 1364 nM vs ^NF54‑K13‑C580Y‑PRC1664^EC_50_^8 h^ = 675 nM; Figure 4B). Consistent with our previous findings using the Dd2 strain,^18^ PRC1697 demonstrated greater potency than PRC1584 in both the WT and mutant parasites following 72 h of continuous exposure (^NF54‑WT‑PRC1697^EC_50_^72 h^ = 59 ± 5 nM vs ^NF54‑WT‑PRC1584^EC_50_^72 h^ = 140 ± 15 nM; ^NF54‑K13‑C580Y‑PRC1697^EC_50_^72 h^ = 38 ± 4 nM vs ^NF54‑K13‑C580Y‑PRC1584^EC_50_^72 h^ = 94 ± 13 nM; Figure 4B and Table S1). Notably, PRC1697 demonstrated approximately 5-fold higher potency than PRC1584 following an 8 h exposure in the NF54-WT strain and exhibited 3-fold greater potency against the NF54-K13-C580Y mutant. Collectively, these findings demonstrate that the PfK13-C580Y mutation confers a distinctive collateral sensitivity profile by increasing susceptibility to PRC1584 and its analogs, further supporting a specific association with the C580Y mutation independent of reduced DHA sensitivity.

### Effects of PRC1584 and Its Analogs on Ring-Stage Parasite Morphology

As shown in Figure 3, PRC1584 affects ring stages within 8 h of exposure. To further investigate whether these effects persist beyond the initial treatment window, we examined the morphological development of the ring stages after drug removal. Highly synchronous ring-stage cultures were treated with either 500 nM PRC1584, PRC1697, PRC1664, or 700 nM DHA for 8 h, after which treatments were removed, and cultures were maintained in drug-free media. A concentration of 500 nM, corresponding to the 8 h EC_50_ value of PRC1584 in NF54-WT, was selected to allow direct potency comparisons across analogs (Figure 4B). Giemsa-stained thin blood smears were prepared from both treated and untreated controls every 24 h for three consecutive days and analyzed by light microscopy. As shown in Figure 5, parasites treated with PRC1584 and PRC1697 exhibited a dormant-like morphology comparable to that observed following DHA exposure, characterized by small parasites with condensed chromatin and reduced cytoplasm. In contrast, consistent with its higher 8 h EC_50_ (Figure 4B), a subpopulation of parasites treated with PRC1664 progressed normally through the intraerythrocytic cycle. Based on the dormant-like morphology identified following short-term exposure, we subsequently investigated whether ring-stage parasites treated with PRC1584 could resume growth over time.

**Figure 5:**
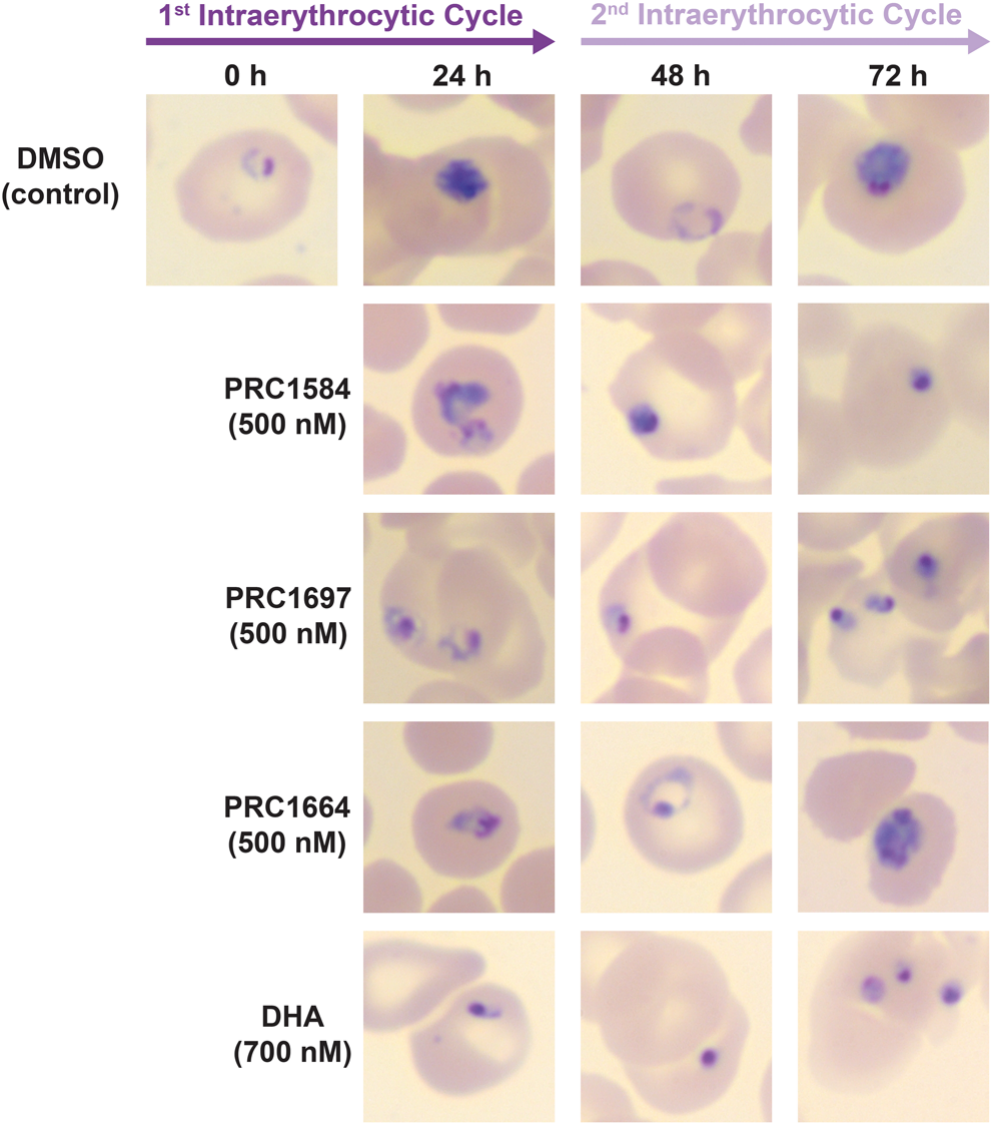
Effect of PRC1584 and its analogs on the morphology of ring-stage parasites. Highly synchronous (6–8 h postinvasion) NF54-WT ring-stage cultures were treated with 500 nM PRC1584, PRC1697, PRC1664 or 700 nM DHA for 8 h. Following drug removal, cultures were maintained in drug-free media and monitored over 3 days by Giemsa-stained thin blood smears and light microscopy. Parasites treated with PRC1584 and PRC1697 displayed dormant-like morphology, characterized by condensed chromatin and reduced cytoplasm, similar to DHA-treated parasites.

### PRC1584 and Its Potent Analog PRC1697 Are Active against Ring Stages

It is well-established that short exposure to DHA induces a dormant state in *P. falciparum* rings, with cultures typically recrudescing 3–4 days after DHA treatment is removed.^30^ To be effective, novel antimalarials should eliminate ring-stage parasites before they enter dormancy or progress to transmissible gametocyte stages. To determine whether parasites exhibiting PRC1584- and PRC1697-induced dormant-like morphology could also recrudesce, we performed ring recrudescence assays following 8 h of exposure to PRC1584 and its analogs (Figure 6A). A concentration of 500 nM, corresponding to the 8 h EC_50_ value of PRC1584 in NF54-WT, was selected to allow direct potency comparisons across analogs (Figure 6A). As expected, after an 8 h treatment with 700 nM DHA followed by washout, recrudescence occurred after 3–5 days in the NF54-WT strain (DHA-sensitive) and within 1–4 days in the NF54-K13-C580Y line (DHA-resistant) (Figures 6B,C and S7). In contrast, cultures treated with 500 nM PRC1584 showed markedly delayed or no recrudescence within 17–30 days post-treatment in both lines. The variation between experiments was expected, as the concentration used was around the 8 h EC_50_ value for NF54-WT strain and twice for the mutant line (Figure 6A). Similar results were observed in the Dd2 and 4G strains, in which higher concentrations prevented recrudescence (Figure S8). Notably, no recrudescence was observed in cultures treated with 500 nM PRC1697, regardless of the presence of the PfK13 mutation (Figure 6B,C). Consistent with its higher 8 h EC_50_ value of 1364 nM for NF54-WT and 675 nM for NF54-K13-C580Y line (Figure 4B and Table S1), parasites exposed to 500 nM PRC1664 resumed growth immediately post-treatment in the NF54-WT strain, whereas a slight delay was observed in NF54-K13-C580Y. Collectively, these findings indicate that an 8 h exposure to PRC1584 or PRC1697 efficiently eliminates ring-stage *P. falciparum* parasites, thereby preventing recrudescence.

**Figure 6:**
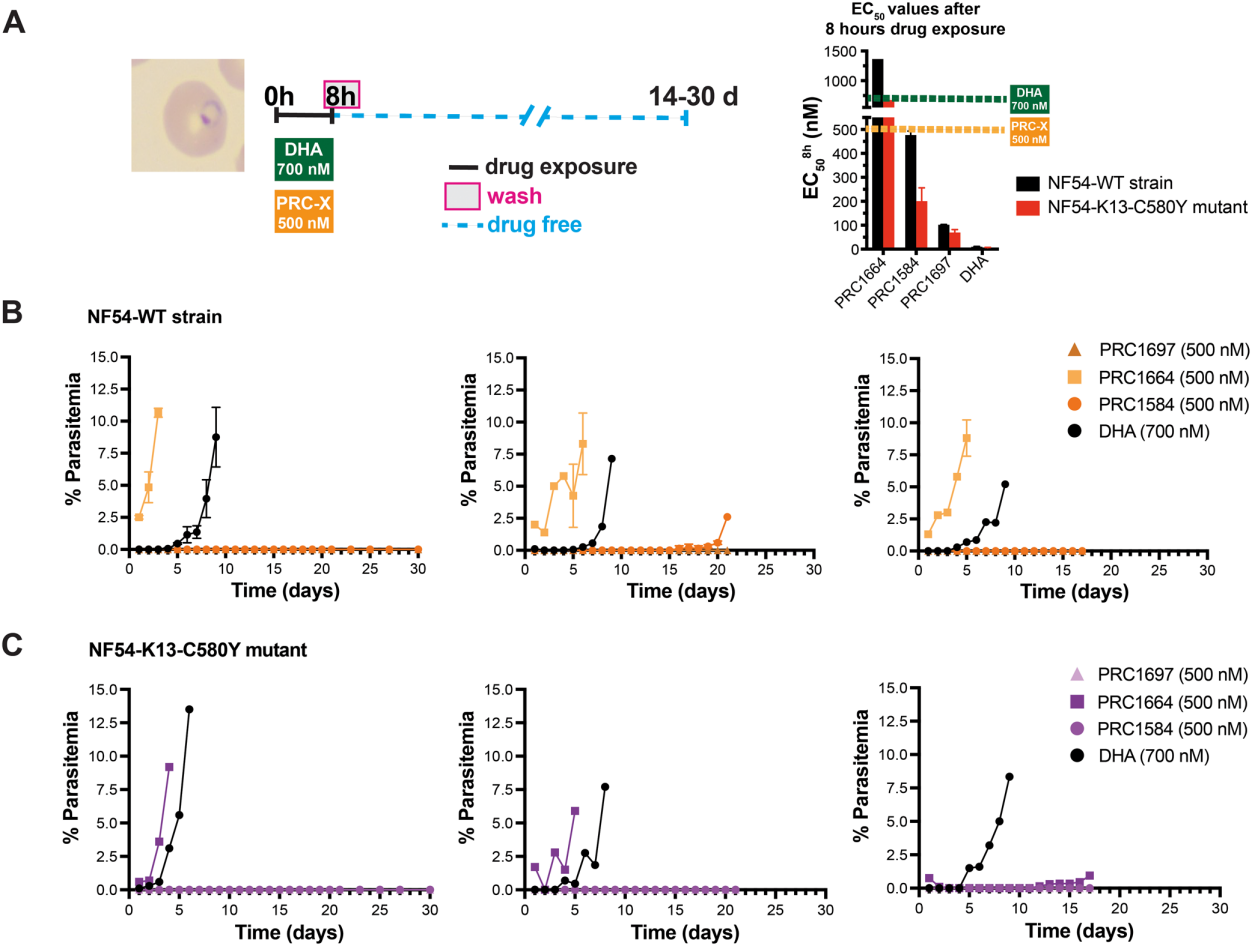
Ring-stage recrudescence following short-term drug exposure. (A) Schematic of the experimental design used to monitor recovery following 8 h of drug exposure in highly synchronous ring-stage cultures (6–8 h postinvasion), as detailed in the Materials and Methods section. Graph depicts the 8 h EC_50_ values as shown in Figure 4B. Giemsa-stained thin blood smears were prepared daily to assess parasite morphology and viability. (B) Recrudescence outcomes for NF54-WT (DHA-sensitive) cultures following treatment with PRC1584, PRC1697, PRC1664, or DHA (control). (C) Recrudescence outcomes for NF54-K13-C580Y (DHA-resistant) cultures under the same treatment conditions. Each panel shows data from a separate experiment, which was independently evaluated by two microscopists under blinded conditions.

### PRC1584 and Its Potent Analog PRC1697 Are Active against DHA-Induced Dormant Parasites

There is increasing evidence suggesting that activity against artemisinin derivative-induced dormant rings should be considered the “gold standard” for evaluating potential partner drugs.^14,16,31,32^ This property could enable the future development of novel ACTs even in the context of ART-R as well as the advancement of new combinations. To assess whether these β-carbolines display activity against DHA-induced dormant parasites, we treated highly synchronous ring cultures (6–8 h postinvasion) with 700 nM DHA for 8 h. Following incubation, drug was removed and incubated in drug-free media for 16 h to ensure that parasites displayed morphological dormancy, at which time these parasites were treated with either PRC1584, PRC1697, PRC1664, or DMSO (Figure 7A). Based on previous studies showing that many antimalarials only delayed recrudescence,^12,16,32^ indicating partial toxicity to persisters, we selected a concentration of 1000 nM (approximately twice the 8 h EC_50_ value for PRC1584 in NF54-WT) for these experiments. Following an initial 8 h exposure to 700 nM DHA (DHA/DMSO), recrudescence occurred at similar time points in both the NF54-WT strain and the PfK13-C580Y mutant line, consistent with earlier observations (Figures 7B,C and S9). Remarkably, when DHA-induced dormant parasites were subsequently treated with PRC1584 or PRC1697, no proliferating parasites were detected for up to 20 days. These findings indicate that both PRC1584 and PRC1697 effectively eliminate DHA-induced dormant parasites regardless of DHA sensitivity (Figure 7B,C). In contrast, treatment with 1000 nM PRC1664 delayed recrudescence by only several days, suggesting partial toxicity to persisters. Furthermore, delayed recrudescence was observed in W2 (artemisinin susceptible) and 4G strains following treatment with PRC1664 or PRC1584, suggesting that factors unique to each strain may affect the compounds’ efficacy against persisters (Figure S10).

**Figure 7:**
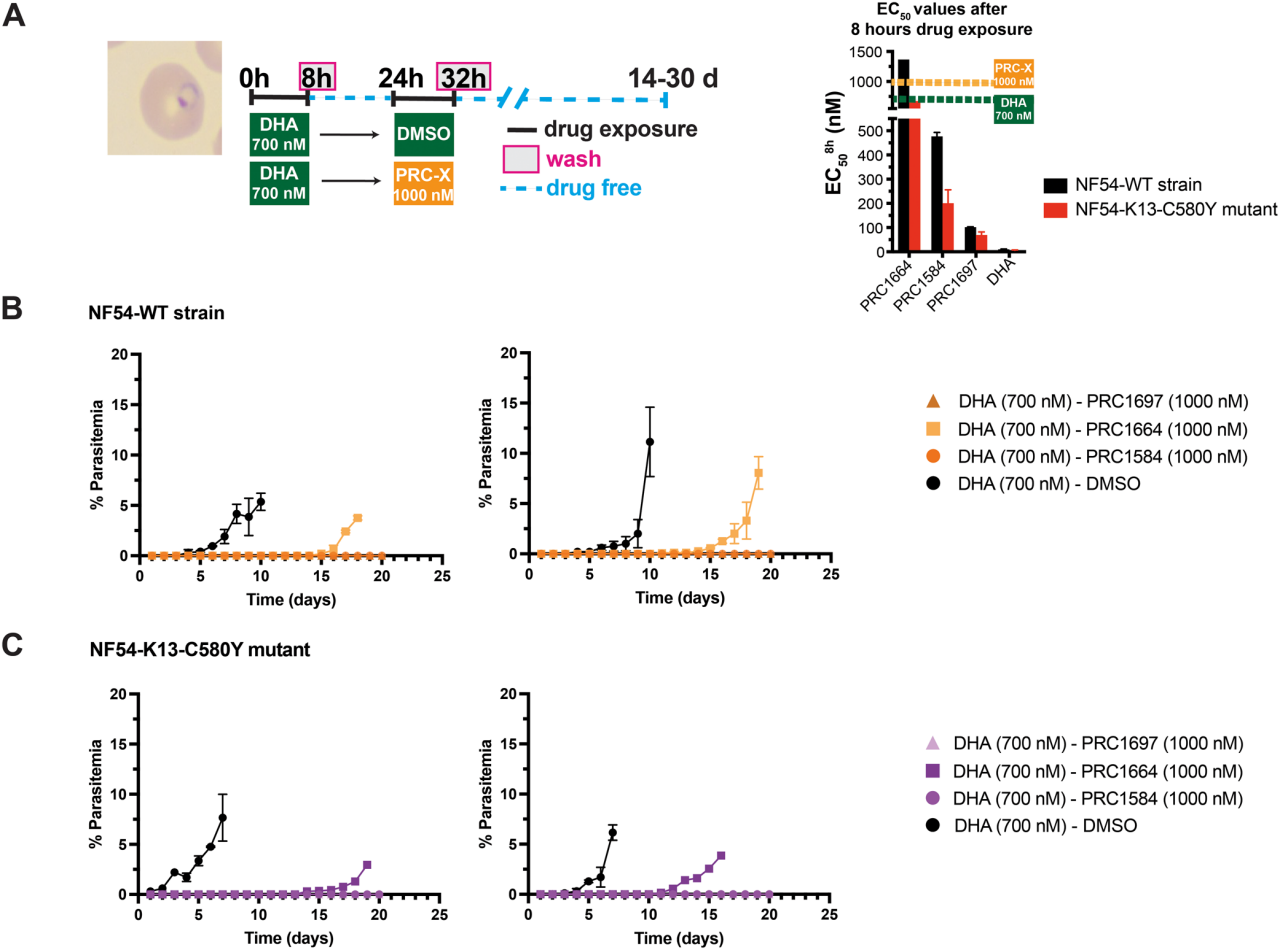
Effect of PRC1584 and related analogs on DHA-induced dormant parasites. (A) Schematic of the experimental design used to monitor recovery following 8 h of drug exposure in DHA-induced dormant *P. falciparum* parasites, as detailed in the Materials and Methods section. Graph depicts the 8 h EC_50_ values as shown in Figure 4B. Giemsa-stained thin blood smears were prepared daily to assess the parasite morphology and viability. (B) Effects of PRC1584 and its analogs (PRC1697 and PRC1664) on DHA-induced dormant parasites in the NF54-WT strain (DHA-sensitive). (C) Recrudescence outcomes for NF54-K13-C580Y (DHA-resistant) cultures under the same treatment conditions. Each panel shows data from a separate experiment, which was independently evaluated by two microscopists under blinded conditions.

## Discussion

Malaria remains a significant global health issue, with increasing drug resistance, including ART-R, threatening current control efforts. A major challenge lies in the parasite’s capacity to survive treatment by entering a dormant state; artemisinin derivatives facilitate this dormancy and fail to fully eliminate it. A recent review described how temporary growth arrest in *P. falciparum* ring-stage parasites, a nongenetic survival mechanism, contributes to ART-R, underscoring the need for therapeutic strategies that can target growth-arrested ring stages, which may underlie treatment failure and persistent infection.^33^ Recent studies describing improved methods for isolating and characterizing *P. falciparum* blood-stage dormant parasites induced by DHA further underscore the importance of effectively studying and targeting these forms.^31^ Dormant parasites exhibit hallmarks of cellular quiescence/senescence and significant drug resilience, providing insights into their capacity to evade current treatments and reinforcing the need for compounds capable of eliminating these populations.^14^ Recognizing that drugs may show differential activity against proliferating ring stages versus quiescent or dormant parasites has led to the development of specialized assays to specifically identify compounds effective against these drug-resilient forms.^12,32^

In this study, we provide a comprehensive characterization of a novel β-carboline class of antimalarials, focusing on PRC1584 and the more potent analog PRC1697.^17,18^ These compounds exhibited compelling activity against both proliferating ring stages and DHA-induced dormant parasites. Our findings show that PRC1584 and PRC1697 exhibit robust activity primarily against the asexual intraerythrocytic stages of *P. falciparum* (Figure 1), with EC_50_ values in the 60–200 nM range after 72 h of drug exposure across various strains with diverse resistance profiles (Table S1).^17,18^ Additionally, PRC1584 showed micromolar activity against gametocytes and gamete formation, but had low activity against *P. berghei* liver schizonts (Figure 1), positioning this class as a target candidate profile 1 (TCP-1) compound.^34^

Refractoriness to resistance selection is a highly desirable characteristic for any new antimalarial,^34^ and a particularly valuable feature of PRC1584 is its remarkable resistance barrier. Rigorous *in vitro* resistance selection experiments employing various constant drug pressure and pulsed-treatment protocols consistently failed to yield resistant parasites with a MIR greater than 10^9^ infected red blood cells, qualifying this compound as “irresistible”.^35^ Furthermore, PRC1584 retained potent activity against a diverse panel of fresh *P. falciparum* clinical isolates from Uganda, with a mean EC_50_ value of 70 nM (range 26–177 nM), consistent with its activity against laboratory strains (Table S1). Combined with previous findings confirming no cross-resistance with existing known mechanisms of resistance, these data underscore the potential utility of this scaffold in endemic regions with prevalent multidrug-resistant parasites.^1,3,5^

Detailed stage-specific assays revealed a monophasic survival profile with the greatest activity during late ring and early trophozoite stages. Remarkably, an 8 h exposure to PRC1584 during the early trophozoite stage yielded an EC_50_ value approaching that of a continuous 72 h exposure (Figure 3 and Table S1). Moreover, PRC1697 demonstrated even greater potency against early rings (Figure 4), again approaching the 72 h EC_50_ value (Table S1). Morphological assessments confirmed that PRC1584 arrested parasites at the early trophozoite stage and PRC1697 at late rings, preventing further development (Figure 5), with a minimum effect on late trophozoite and schizont stages (Figure 3A). Biphasic survival curves observed in later stages (Figure 3B) suggest that PRC1584 may act on two distinct molecular targets that may be either functionally related or independent. The molecular target(s) of this class appear to be vital for biological processes during rings and early trophozoite stages, with lower activity in late trophozoites and schizonts, in which a second target may be active, leading to the biphasic response observed. Importantly, the unique asexual blood-stage-specific profile of PRC1584 did not match that of any stage-specific profiles of the 36 clinical or experimental antimalarials profiled by Murithi and colleagues,^24^ suggesting a distinct, potentially novel mechanism of action. The refractoriness to resistance of this class is a major plus for clinical development, but it comes with the disadvantage of preventing ready determination of the mechanism of action.^35^ Currently, we are conducting chemoproteomics studies aimed at identifying potential molecular targets of this novel class.

An especially compelling and therapeutically significant finding is the unique collateral sensitivity observed in the PfKelch13-C580Y background (Figure 4). ART-R, characterized by delayed clearance and reduced drug susceptibility in ring stages, is associated with mutations in the *pfkelch13* gene, with C580Y being the most prevalent mutation in Southeast Asia.^3,10^ Strikingly, the C580Y mutation conferred significantly increased susceptibility to PRC1584 (Figure 4), with approximately 3-fold lower EC_50_ values in the 4G strain (PfKelch13-C580Y) compared to the Dd2 strain (PfKelch13-WT). This collateral sensitivity was confirmed in an isogenic NF54 background and was not observed with other PfKelch13 (R539T) or Pfcoronin (R100 K-E107V) mutations, suggesting a specific association with the C580Y mutation independent of reduced DHA sensitivity. A collateral drug sensitivity phenomenon has been described in *P. falciparum* before, especially in the context of multidrug-resistance protein 1 (PfMDR1).^36^ We recently reported the PfMDR1-G293V mutation as a key mediator of stereospecific resistance to the tetrahydro-β-carboline antimalarial PRC1590 while simultaneously conferring collateral sensitivity to other antimalarials.^37^ Such findings highlight opportunities for drug cycling and combination therapies.

Beyond their activity against actively proliferating ring-stage parasites, PRC1584 and PRC1697 were highly active against DHA-induced dormant parasites. It is well-established that short exposure to DHA induces a dormant state in *P. falciparum* rings, with cultures typically recrudescing 3–4 days after DHA treatment removal.^30^ A recent study has shown that this dormant state displays hallmarks of cellular quiescence/senescence and drug resilience, with a unique transcriptional profile without resemblance to that of any normal parasite stages.^14^ Our morphological assessments initially revealed that parasites treated with PRC1584 and PRC1697 displayed dormant-like features comparable to those observed following DHA exposure (Figure 5), characterized by small parasites with condensed chromatin and reduced cytoplasm. However, we conducted ring-stage recrudescence assays, and under this assay’s conditions, exposure to 700 nM DHA for 8 h typically resulted in parasite reemergence in culture within days (Figure 6), while exposure to 500 nM PRC1584 for only 8 h either significantly delayed or completely prevented recrudescence for up to 30 days, regardless of DHA sensitivity. The variation between experiments was expected, as the concentration used was around the 8 h EC_50_ value for the NF54-WT strain and twice that for the mutant line (Figure 6A). This ability to prevent recrudescence from ring stages underscores the therapeutic potential of PRC1584 and PRC1697. Targeting early ring stages is a highly desirable attribute for antimalarials, as these forms may progress to either asexual or sexual (transmission) blood stages, or alternatively enter dormancy under adverse conditions.^33^ Furthermore, identification of dormant parasite forms triggered by drug exposure during the asexual intraerythrocytic life cycle has recently gained attention, and its molecular characteristics are only beginning to be understood.^14^ Therefore, further research is necessary to determine at the molecular level whether PRC1584 and its analogs induce dormancy equivalent to that associated with artemisinins.

Currently, there are no treatments available that specifically target the dormant blood stages of *P. falciparum*. Notably, when specifically tested against DHA-induced dormant parasites, both PRC1584 and PRC1697 completely eliminated parasites, with no recrudescence observed for up to 20 days, irrespective of DHA sensitivity (Figure 7). In contrast, PRC1664 only delayed recrudescence, thus showing partial toxicity to persisters. Given that parasite growth typically resumes within 3–5 days following DHA withdrawal, and β-carboline analogs were administered for only 8 h starting 16 h post-DHA treatment (Figure 7A), the observed lack of recrudescence is unlikely to be attributable solely to effects on the proliferating ring-stage parasites. Presumably, the mechanism by which the novel compounds kill DHA-induced dormant parasites is related to their toxicity to proliferating ring stages, and thus, the lower asexual blood-stage potency of PRC1664 relative to PRC1584 and PRC1697 may come into play. It is also possible that differences in lipophilicity may affect the ability of compounds to accumulate in DHA-induced dormant parasites. As expected from the ethanolamine group present in PRC1664, it is less lipophilic than PRC1584 and PRC1697 (calculated Log D values of 3.3, 3.5, and 4.1, respectively, CDD Vault, Collaborative Drug Discovery). This capability to kill asexual blood-stage dormant forms is a major advantage and positions this class as an ideal candidate for inclusion in future artemisinin-based combination therapies. Nevertheless, confirmation of the ability of PRC1584 and PRC1697 to clear dormant parasites *in vivo* in relevant animal models is essential. In addition, our findings align with the growing body of research demonstrating the importance of targeting dormant stages, highlight the utility of specialized approaches for identifying such active compounds, and reinforce the crucial need to better characterize dormant parasites.^16,31,32^

Interestingly, the spiroindolone KAE609, which targets PfATP4, does not induce dormant-like parasites but prevents recrudescence of DHA-induced dormant parasites.^38^ Differently from PRC1584, KAE609 does not affect ring stages but is effective against early trophozoites and later developmental stages.^24^ Thus, our data suggest that PRC1584 operates via a different mechanism than KAE609. Likewise, imidazolopiperazines such as GNF179 effectively kill both ring stages and DHA-induced dormant parasites in wildtype and K13-mediated artemisinin-resistant *P. falciparum* strains.^39^ However, imidazolopiperazines may act through mechanisms different from those of PRC1584, as they are cidal against schizonts, whereas PRC1584 does not prevent merozoite formation (Figure 3A). Therefore, it is likely that this class of β-carboline acts against distinct molecular targets in proliferating ring-stage parasites compared to those in DHA-induced dormant parasites.

In summary, our study highlights a novel β-carboline class with a high barrier to resistance, potent activity against ring stages, and the ability to eliminate DHA-induced dormant parasites. These attributes directly address critical challenges in malaria chemotherapy.^34^ In addition, the unique collateral sensitivity to PfKelch13-C580Y mutation offers a strategic advantage, providing a potential therapeutic avenue to combat artemisinin resistance. Importantly, the proven capacity of these compounds to eradicate DHA-induced dormant parasites underscores their promise as optimal partner drugs for next-generation ACTs. Continued research to identify specific molecular targets and refine this scaffold will be crucial to achieving this goal.

## Materials and Methods

### *P. falciparum* Growth Inhibition Assay

Dose-dependent growth inhibition with the reported compounds was evaluated using a 10-point dilution series and the *in vitro* SYBR Green I assay as a readout. Synchronous ring-stage parasites (1% starting parasitemia and 1% hematocrit) were cultured in 96-well half-area dark plates and continuously exposed to each compound for 72 h at 37 °C under reduced oxygen conditions (5% CO_2_, 5% O_2_, and 90% N_2_). After 72 h, parasite growth was assessed by the SYBR Green I assay as previously described.^40^ SYBR Green I was excited at 485 nm, and its emission was measured at 535 nm using a Cytation5 plate reader (Agilent Bio Tek). Parasite growth was normalized to that of untreated control parasites and calculated as a percentage. The background was determined by using uninfected red blood cells (RBCs).

Dose-dependent assays were performed in at least two biological replicates and two technical replicates. Reported values represent the mean of biological replicates with the standard error of the mean (SEM). Initial concentrations were optimized after compound screening to ensure the EC_50_ value fell within the tested range. DMSO concentrations were maintained at ≤0.02% in all assays. Data were fitted using a four-parameter logistic dose–response curve, and half-maximal effective concentration (EC_50_) values were calculated using GraphPad Prism (GraphPad Software, Inc.). Assays comparing EC_50_ values across different parasite lines were performed concurrently.

### Dose-Dependent Stage-Specificity Assays

Stage-specific assays were performed as previously described^24^ with slight modifications.^37^ Parasites of the *P. falciparum* 3D7 strain were synchronized in the ring stage 48 h prior to the assay by two rounds of 5% sorbitol treatment (Sigma-Aldrich, St. Louis, MO) with a 6 h interval between treatments. After parasites completed one life cycle and reinjected RBCs, an additional sorbitol treatment was performed on the day of the assay to obtain highly synchronous early rings. Synchronized infected RBCs were plated in five 96-well plates and sequentially exposed to compounds for 8 h windows starting at the following developmental stages: early rings (0–8 h), late rings (8–16 h), early trophozoites (16–24 h), late trophozoites (24–32 h), and schizonts (32–40 h). After each exposure window, compounds were removed by three rounds of washing with prewarmed RPMI to avoid growth delays, and infected RBCs were transferred to a new plate. Plates were incubated under standard culture conditions, and parasite growth was assessed 72 h after the start of the assay using the SYBR Green I assay as described above. A 72 h continuous exposure dose–response curve was included in parallel as control. Dose–response data were fitted using a four-parameter logistic dose–response curve or, where appropriate, a biphasic dose–response, and EC_50_ values were calculated using GraphPad Prism (GraphPad Software, Inc.). Assays were performed in at least two independent biological replicates, each with two–four technical replicates.

A similar experimental design was used to determine the EC_50_ values in ring stages following 6 or 8 h exposure periods in a dose-dependent manner using different strains and mutants. Assays comparing EC_50_ values across different parasite lines were performed concurrently. These assays were performed in one or two independent biological replicates, each with two–four technical replicates.

### Modified Recrudescence and DHA-Induced Dormancy Survival Assays

Recrudescence was monitored in bulk cultures as previously described,^12^ with slight modifications (Figures 6A and 7A). Parasites were synchronized twice with 5% sorbitol with a 6 h interval between treatments. Thirty-six hours after the final synchronization, 5 mL cultures of early ring stages (0–6 h postinvasion) were adjusted to 3% parasitemia and 5% hematocrit and treated with DHA (700 nM), PRC1584, PRC1697, or PRC1664 (500 nM) for 8 h at 37 °C with shaking under reduced oxygen conditions (5% CO_2_, 5% O_2_, and 90% N_2_). Following incubation, parasites were washed three times with prewarmed complete RPMI media and transferred to new flasks containing drug-free media. Thin blood smears were prepared every 24 h over a 15–30-day period, fixed with 100% methanol (Sigma-Aldrich, St. Louis, MO), and stained with 20% Giemsa for 15 min (Sigma-Aldrich; diluted in deionized water). After smear preparation, cultures were immediately gassed and returned to 37 °C with shaking. Media was replaced every other day, and 50 μL of fresh blood was added weekly. Recrudescence was assessed by light microscopy, scoring the proportion of viable parasites with a normal morphology following drug removal. For each condition, 10,000 erythrocytes were independently evaluated by two microscopists under blinded conditions, and the results were reported as percentages.

The effect of PRC analogs on DHA-induced dormant parasites was assessed as previously described,^12^ with slight modifications. Bulk cultures were synchronized and treated with 700 nM DHA for 8 h, as described above. At 24 h post-treatment, parasites were treated with 1 μM PRC1584, PRC1697, PRC1664, or DMSO (mock) for 8 h. Final DMSO concentrations did not exceed 0.02%. After incubation, parasites were washed three times with prewarmed complete RPMI media and transferred to new flasks containing drug-free media. Recrudescence was monitored by light microscopy, as described above, for up to 30 days.

### *Ex Vivo* EC_50_ Values against *P. falciparum* Field Isolates in Uganda

The activity of PRC1584 was tested against fresh clinical *P. falciparum* isolates using a 72 h growth inhibition assay with parasite DNA detection by SYBR Green I as previously described.^40,41^ These isolates were collected in June and July 2018 from patients living in the Tororo and Busia Districts, Uganda, who were newly diagnosed with *P. falciparum* malaria before antimalarial treatment was administered. The studies were approved by the Uganda National Council of Science and Technology, the Makerere University Research and Ethics Committee (SBS-REC 341), and the University of California San Francisco Committee on Human Research (16-19084).

## Supplementary Material



## Notes

Schemes of *in vitro* selection of drug resistance (Figure S1); dose-dependent response curves of PRC1584 in the 3D7 strain (Figure S2); dose-dependent response curves used to calculate the 8 h EC_50_ values represented in Figure 4 (Figure S3); collateral drug sensitivity to PRC1584 after 6 h versus 72 h exposure in PfK13-C580Y mutant parasites (Figure S4); collateral drug sensitivity to PRC1584 after 6 h versus 72 h exposure in Kelch-13 R539T mutant parasites (Figure S5); dose-dependent response curves for Pikine-WT strain and Pikine-Pfcoronin-R100 K-E107V mutant line after 6 and 8 h exposure to PRC1584 (Figure S6); initial ring-stage recrudescence assays of parasites exposed to PRC1584 performed before PRC1664 and PRC1697 were designed and synthesized (Figure S7); ring-stage recrudescence assays with varying concentrations of PRC1584 (Figure S8); effect of PRC1584 and PRC1664 on DHA-induced dormant parasites (Figure S9); initial studies evaluating the effect of PRC1584 and PRC1664 on DHA-induced dormant parasites in W2 strain and 4G-K13-C580Y mutant strain (Figure S10); summary of *in vitro* potencies (Table S1); and additional Materials and Methods (PDF)

## ⧓

This paper is dedicated to the memory of Roland Cooper
