# Supplementary material for: Characterization of β‑Carboline Derivatives Reveals a High Barrier to Resistance and Potent Activity against Ring-Stage and DHA-Induced Dormant Plasmodium falciparum
Source: ACS Infect Dis. 2025 Oct 17;11(11):3310–22. doi: 10.1021/acsinfecdis.5c00714 (PMC12624827; doi:10.1021/acsinfecdis.5c00714)
Supplement: Supplementary file 1 [file id5c00714_si_001.pdf]

Supporting Information

Characterization of β-carboline Derivatives Reveals a High Barrier to Resistance and Potent Activity Against Ring-Stage and DHA-Induced Dormant *Plasmodium falciparum*

Reagan S. Haney[1,2], Joshua H. Butler[1,2], Lyric A. Wardlaw[1,2], Emilio F. Merino[1,2], Victoria Mendiola[2,8], Caitlin A. Cooper[2], Jopaul Mathew[3], Patrick K. Tumwebaze[4], Philip J. Rosenthal[5], Roland A. Cooper[6#], Dennis E. Kyle[2,7,8], Zaira Rizopoulos[9], Delphine Baud[9], Stephen Brand[9], Maxim Totrov[10], Paul R. Carlier[3,11], Maria Belen Cassera[1,2*]

[1]Department of Biochemistry and Molecular Biology, University of Georgia, Athens, Georgia 30602, United States
[2]Center for Tropical and Emerging Global Diseases, University of Georgia, Athens, Georgia 30602, United States
[3]Department of Chemistry, Virginia Tech, Blacksburg, Virginia 24061, United States
[4]Infectious Diseases Research Collaboration, Kampala, Uganda
[5]Department of Medicine, University of California, San Francisco, California, United States
[6]Department of Natural Sciences and Mathematics, Dominican University of California, San Rafael, California, United States
[7]Department of Cellular Biology, University of Georgia, Athens, Georgia 30602, United States
[8]Department of Infectious Diseases, University of Georgia, Athens, Georgia 30602, United States
[9]Medicines for Malaria Venture, Geneva 1215, Switzerland
[10]MolSoft LLC, San Diego, California 92121, United States
[11]Department of Pharmaceutical Sciences, University of Illinois Chicago, Chicago, Illinois 60612, United States
*Correspondence: maria.cassera@uga.edu
#*This paper is dedicated to the memory of Roland Cooper*

Table of Contents

Figure S1	Page S2
Figure S2	Page S2
Figure S3	Page S3
Figure S4	Page S4
Figure S5	Page S5
Figure S6	Page S6
Table S1	Page S7
Figure S7	Page S8
Figure S8	Page S9
Figure S9	Page S10
Figure S10	Page S11
Materials and Methods	Page S12
References	Page S16

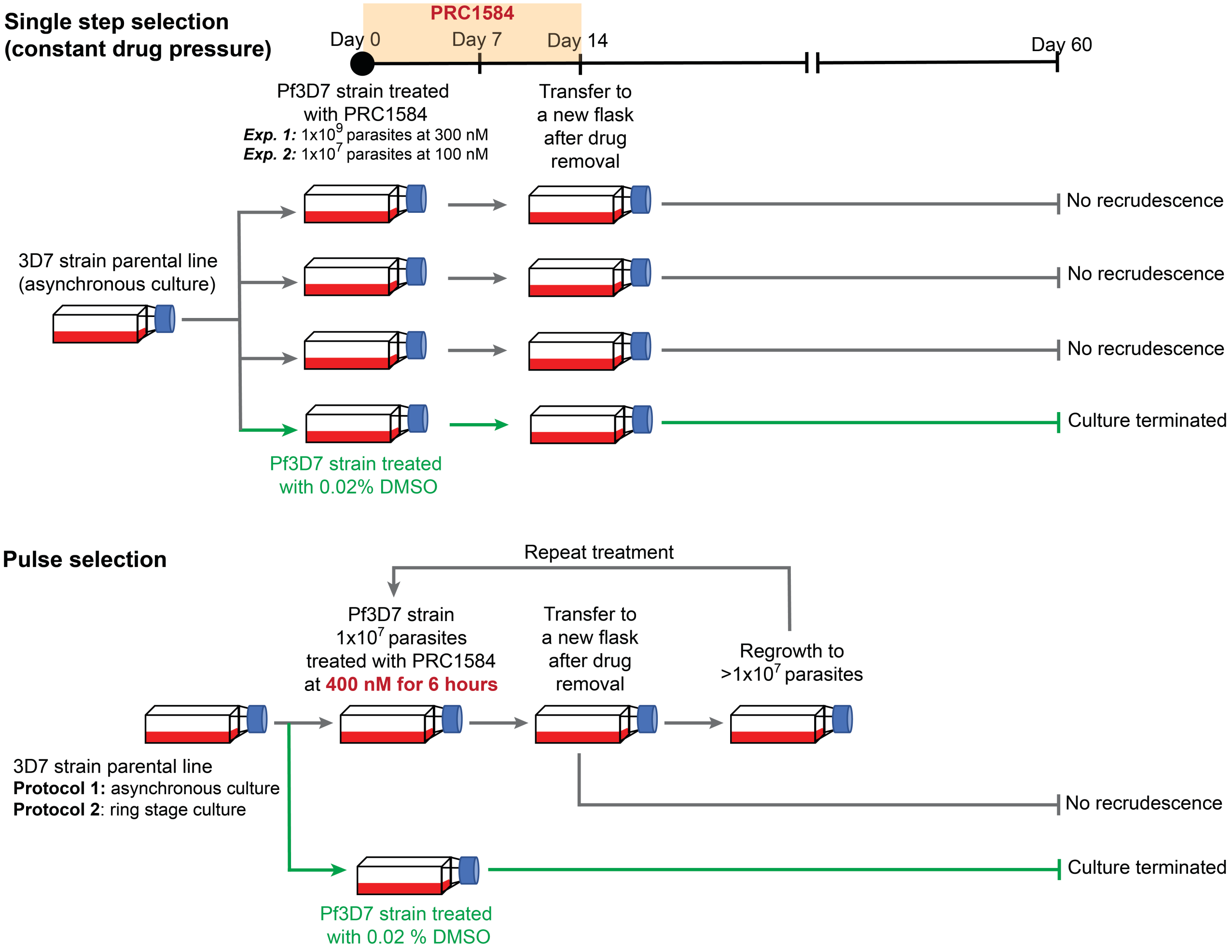

Figure S1. A schematic representation of the *in vitro* resistance selection methodologies employed is shown. Specific details regarding each approach are outlined in the methods section below.

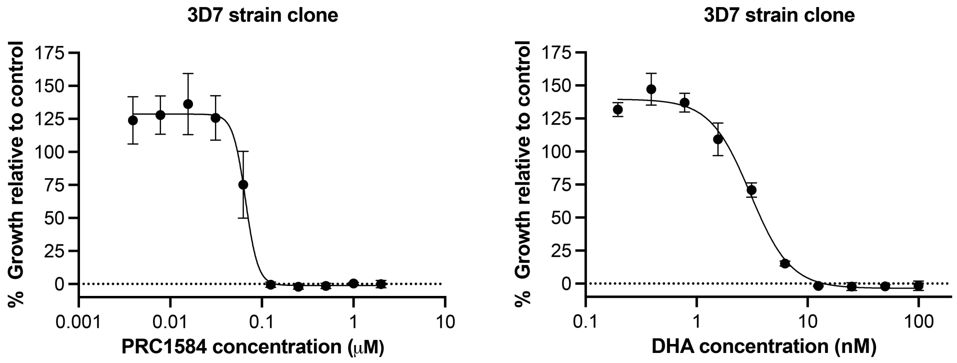

Figure S2. Dose-response curves for PRC1584 and DHA in the 3D7 strain clone (72 hours continuous exposure). Dose-dependent response curves for PRC1584 and DHA (control) were obtained using the *P. falciparum* 3D7 strain clone employed in resistance selection experiments. Data represent the mean from two independent biological replicates; each performed in technical duplicates. All x-axes are displayed in logarithmic scale.

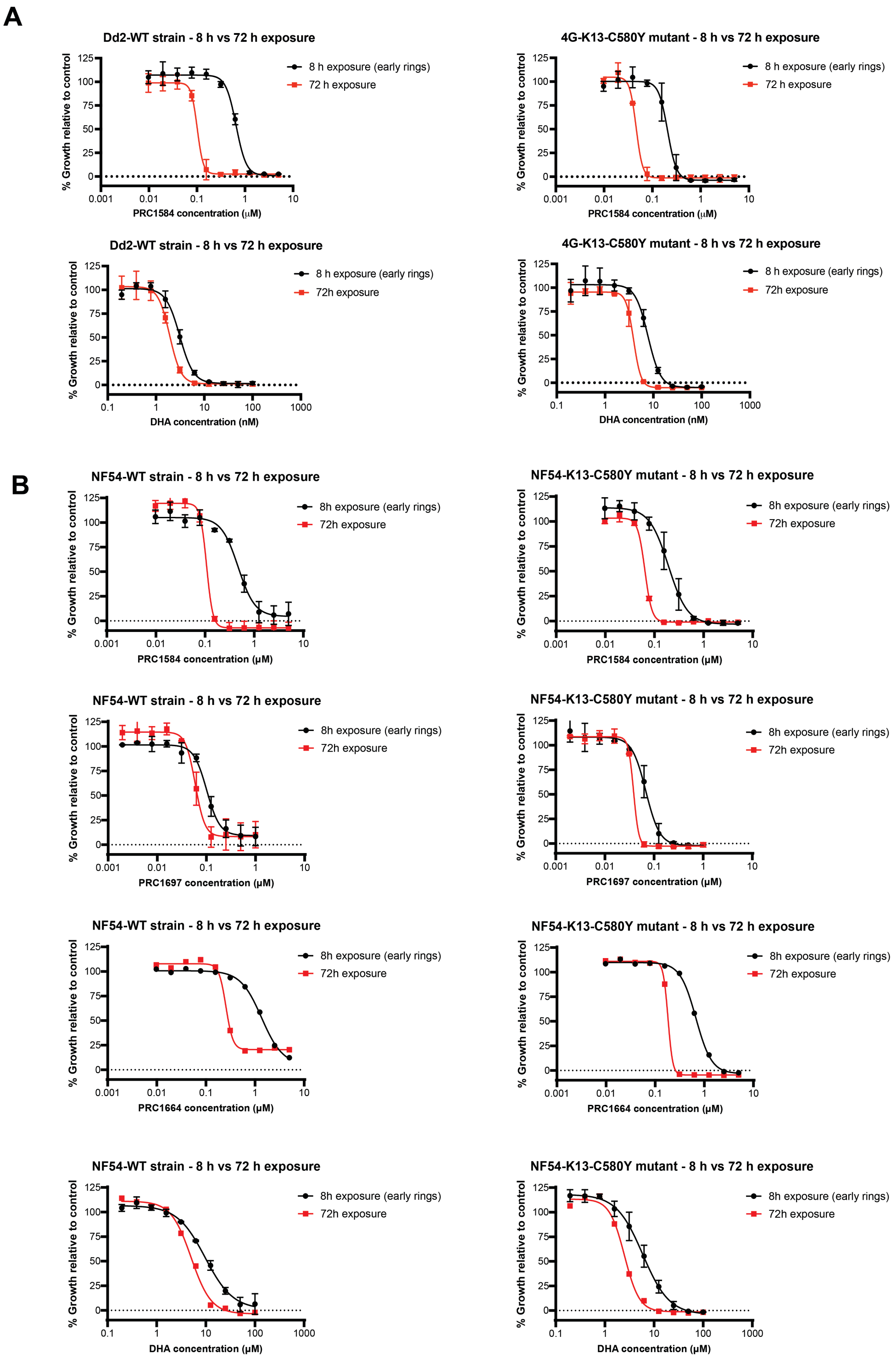

Figure S3. Dose-response curves for Kelch 13 WT and C580Y mutant strains after 8-hour versus 72-hour exposure. **A)** Dose-dependent response curves for the Dd2-WT strain (left) and the 4G-K13-C580Y mutant (right) after 8 hours of exposure (black line) or continuous 72-hour exposure (red lines) to PRC1584 (top row) and DHA (bottom row). **B)** Dose-dependent response curves for NF54-WT strain and NF54-K13-C580Y mutant after 8 hours or 72 hours of exposure to PRC1584 (top row), PRC1697 (second row), and PRC1664 (third row), and DHA (bottom row). These data were used to calculate EC_{50} values shown in Figure 4. Curves represent the mean from one to three biological replicates; each performed in technical triplicates. All x-axes are displayed in logarithmic scale.

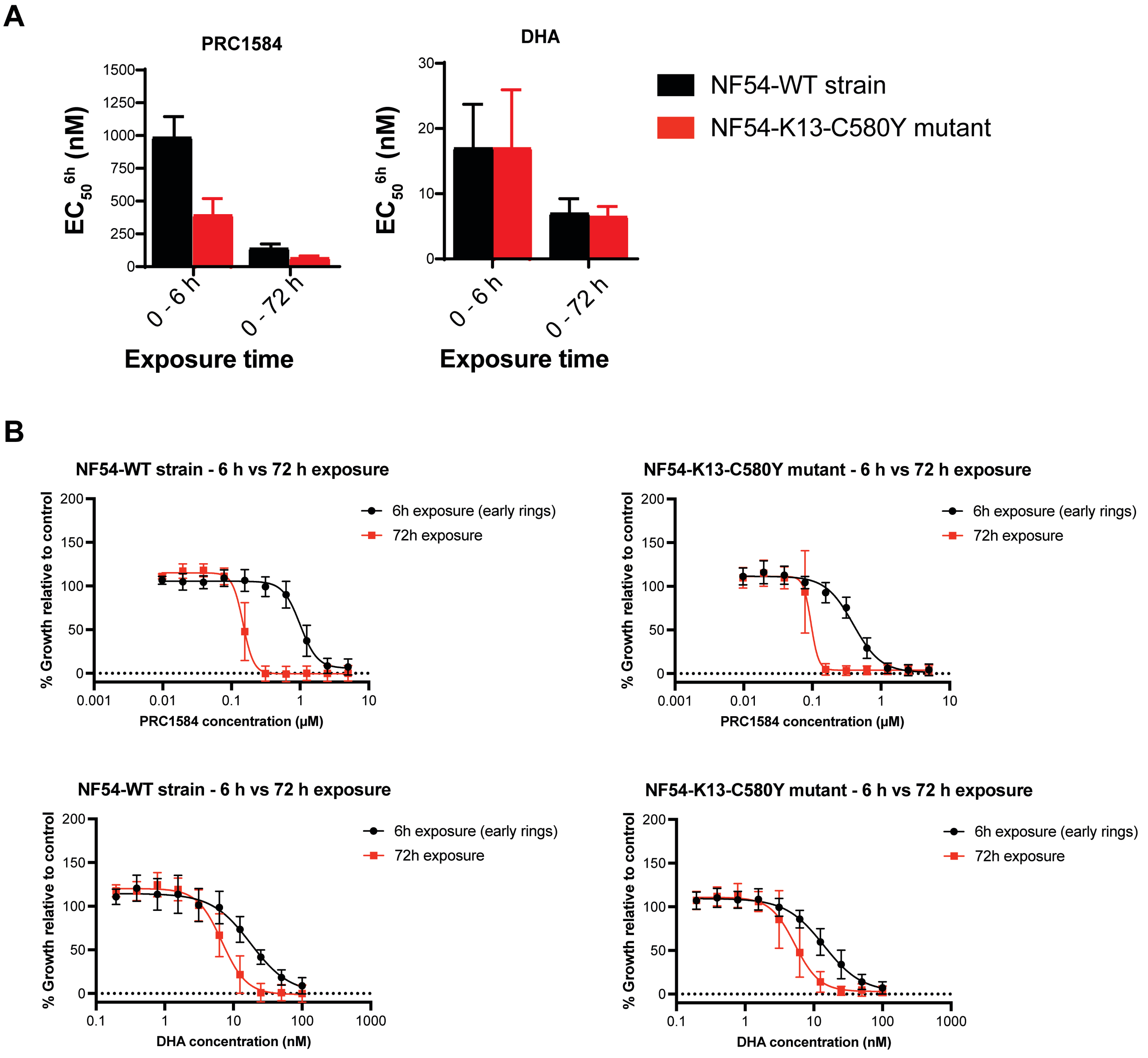

Figure S4. Collateral drug sensitivity to PRC1584 after 6-hour versus 72-hour exposure in PfK13-C580Y mutant. **A)** EC_{50} values for PRC1584 (left) and DHA (right) after 6 hours of exposure (0-6 h, early rings) or continuous 72-hour exposure in NF54-WT (black bars) and NF54-K13-C580Y mutant (red bars) strains. **B)** Dose-dependent response curves for PRC1584 (top) and DHA (bottom) comparing 6-hour exposure (black lines) and 72-hour continuous exposure (red lines) in NF54-WT (left) and NF54-K13-C580Y mutant (right) strains. Data represent the mean ± SEM from six independent biological replicates, each performed in technical triplicates. X-axes are shown on a logarithmic scale.

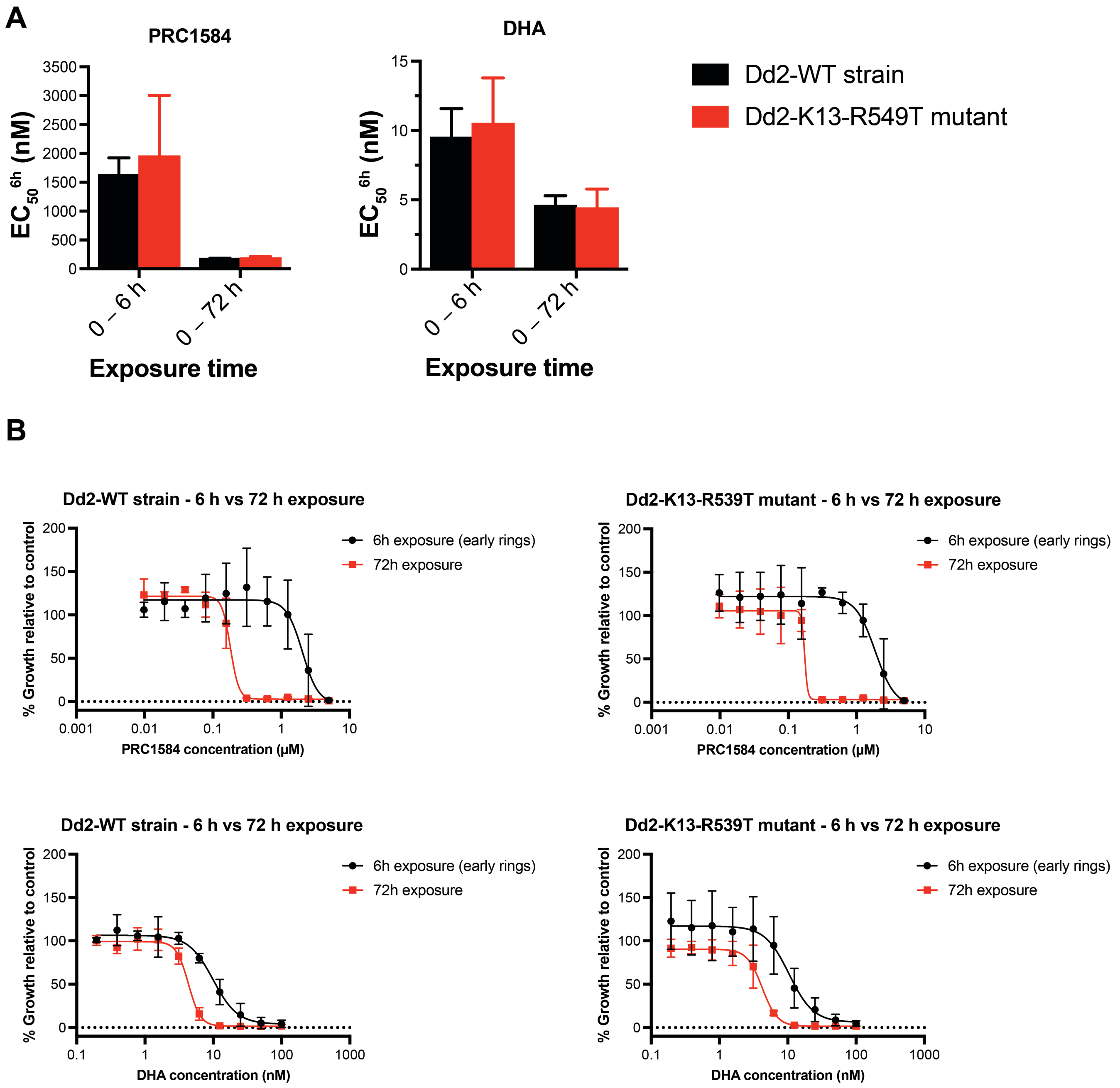

Figure S5. The Kelch-13 R539T mutation does not confer collateral drug sensitivity to PRC1584. **A)** EC_{50} values for PRC1584 (left) and DHA (right) after 6 hours (0–6 h, early rings) or continuous 72-hour exposure in Dd2-WT (black bars) and Dd2-K13-R549T mutant (red bars) strains. Increased sensitivity to PRC1584 in the PfK13-R549T mutant was not observed. **B)** Corresponding dose-dependent response curves for Dd2-WT strain and Dd2-K13-R549T mutant parasites exposed for 6 hours (black lines) or 72 hours (red lines) to PRC1584. The data represent the mean ± SEM from two to three biological replicates, each performed in technical triplicates. All x-axes are shown on a logarithmic scale.

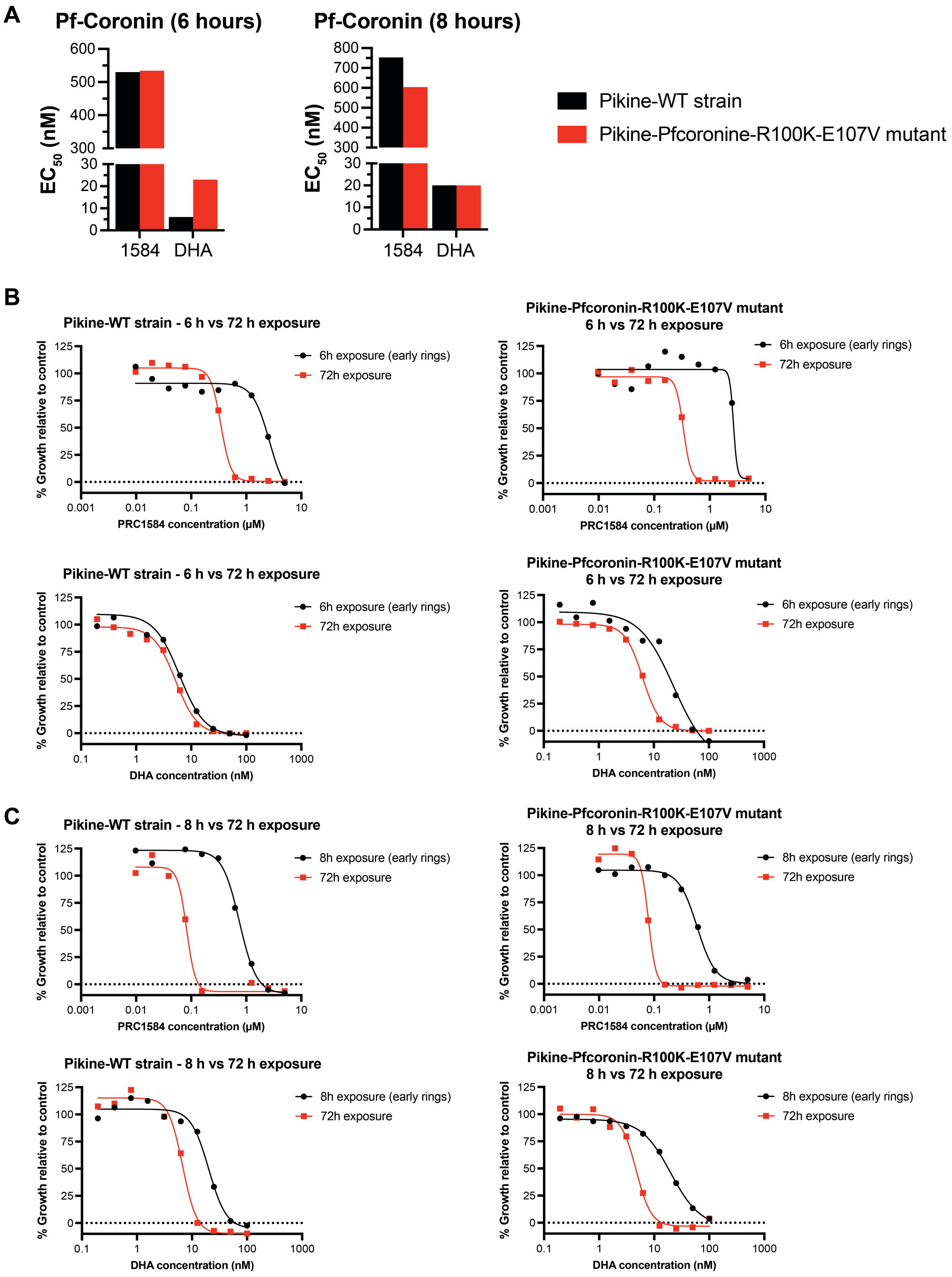

Figure S6. Dose-dependent response curves for Pikine-WT strain and Pikine-Pfcoronin-R100K-E107V mutant line after 6-hour and 8-hour exposure to PRC1584. **A)** EC_{50} values for PRC1584 and DHA after 6 or 8 hours of exposure (early rings) in Pikine-WT (black bars) and Pikine-Pfcoronin-R100K-E107V mutant (red bars) lines. **B)** Dose-response curves for Pikine-WT (left) and Pikine-Pfcoronin-R100K-E107V mutant (right) parasites after 6 hours of exposure (black lines) compared with 72 hours of continuous exposure (red lines) to PRC1584 (top row) or DHA (bottom row). **B)** Dose-response curves corresponding to the 8 hours of exposure to PRC1584 and DHA (control). The data represent the mean from one biological replicate performed in technical triplicates. All x-axes are shown on a logarithmic scale.

Table S1. Summary of the *in vitro* potencies (EC_{50} values; nM) of PRC1584, PRC1967, and PRC1664 across various wild-type strains and mutants at different exposure times

	PRC1584			PRC1697			PRC1664			DHA		
	6 h (early ring)	8 h (early ring)	72 h	6 h (early ring)	8 h (early ring)	72 h	6 h (early ring)	8 h (early ring)	72 h	6 h (early ring)	8 h (early ring)	72 h
3D7		740 ± 60	67 ± 5									3.0 ± 0.3
Dd2*			182 ± 18							10 ± 0.4		4.3
Dd2-R549T	1950 ± 610		188 ± 17							11 ± 2		4.2
Dd2**	1630 ± 170	696 ± 62	114 ± 9			54 ± 8			211 ± 41		3.0 ± 0.2	2.02 ± 0.02
4G-K13-C580Y		211 ± 55	66 ± 35								7.7 ± 1.1	3.9 ± 0.2
W2			100 ± 8									4.7 ± 1.3
NF54	987 ± 64	476 ± 16	140 ± 15		102 ± 2	59 ± 5		1364	256	17.± 3	10 ± 2	7 ± 1
NF54-K13-C580Y	394 ± 51	200 ± 56	94 ± 13		70 ± 13	38 ± 4		675	182	17 ± 4	6 ± 2	5 ± 1
Pikine	530	753	77 ± 7		159	55				6	20	6 ± 1
Pikine-Pfcoronin-R100K-E107V	534	604	73 ± 6		116	37				23	20	6 ± 0.5

Assays comparing EC_{50} values across different parasite lines were performed concurrently.
(*) EC_{50} value obtained concomitant with that of Dd2-R549T.
(**) EC_{50} value obtained concomitant with that of 4G-K13-C580Y.
Gray cells indicate that EC_{50} values were not determined.
The mean and standard error of the mean (SEM) are presented for experiments conducted with two or more independent biological replicates.

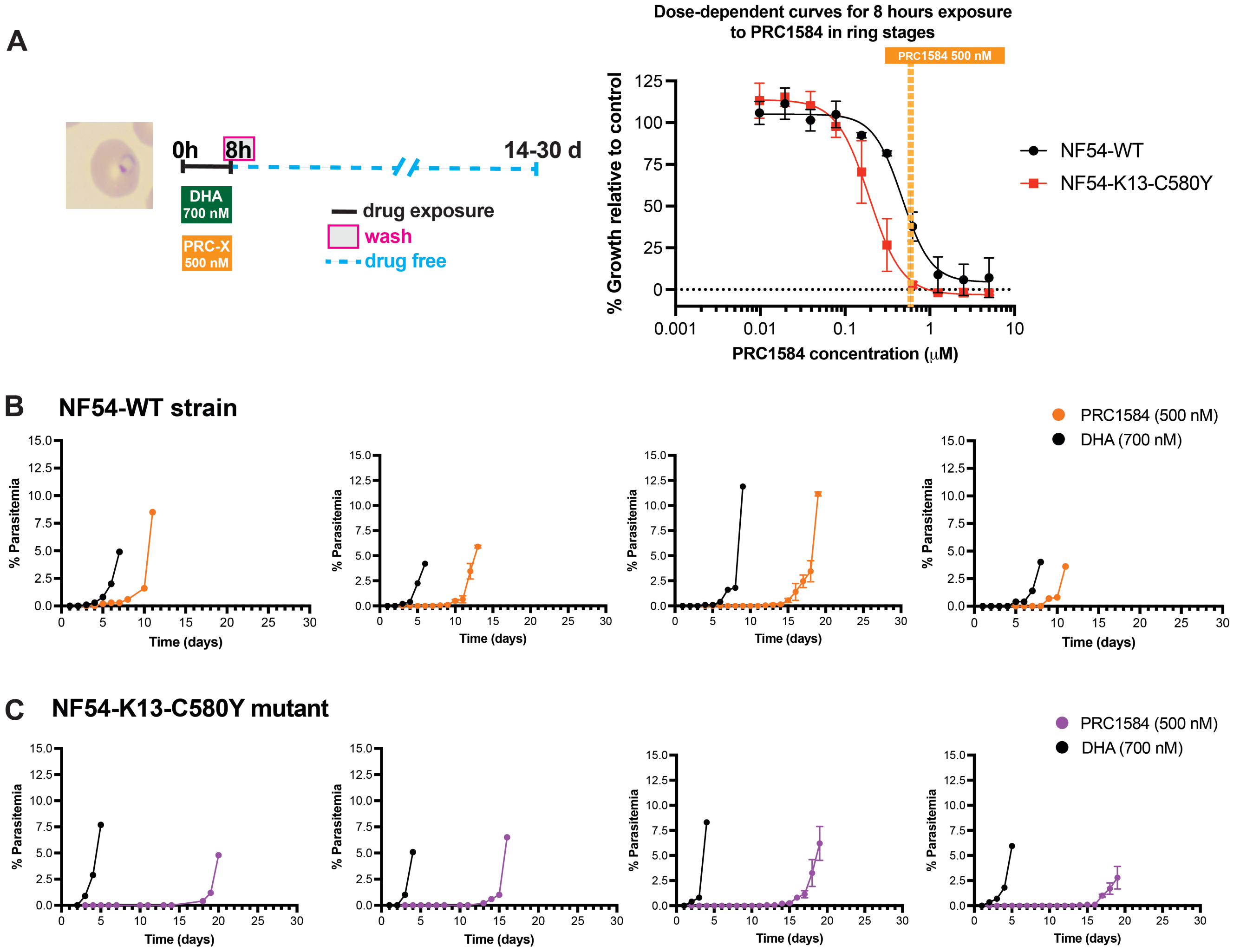

Figure S7. Initial ring stage recrudescence assays of parasites exposed to PRC1584 performed before PRC1664 and PRC1697 were designed and synthesized. **A)** Schematic of the experimental design used to monitor parasite recovery following 8 hours of drug exposure in highly synchronous ring-stage cultures (6-8 h post-invasion). After 8 hours of treatment with 500 nM PRC1584 or 700 nM DHA, cultures were washed and maintained in drug-free medium. Giemsa-stained thin blood smears were prepared daily to track parasite morphology and growth. X-axis is shown on a logarithmic scale for the dose-dependent curves obtained after 8 hours of exposure to PRC1584. **B)** Recrudescence profiles for NF54-WT (DHA-sensitive) cultures treated with PRC1584 (orange) or DHA (black). **C)** Recrudescence profiles for NF54-K13-C580Y (DHA-resistant) cultures under the same treatment conditions (PRC1584, purple; DHA, black). Each panel shows data from a separate experiment, which was independently evaluated by two microscopists under blinded conditions.

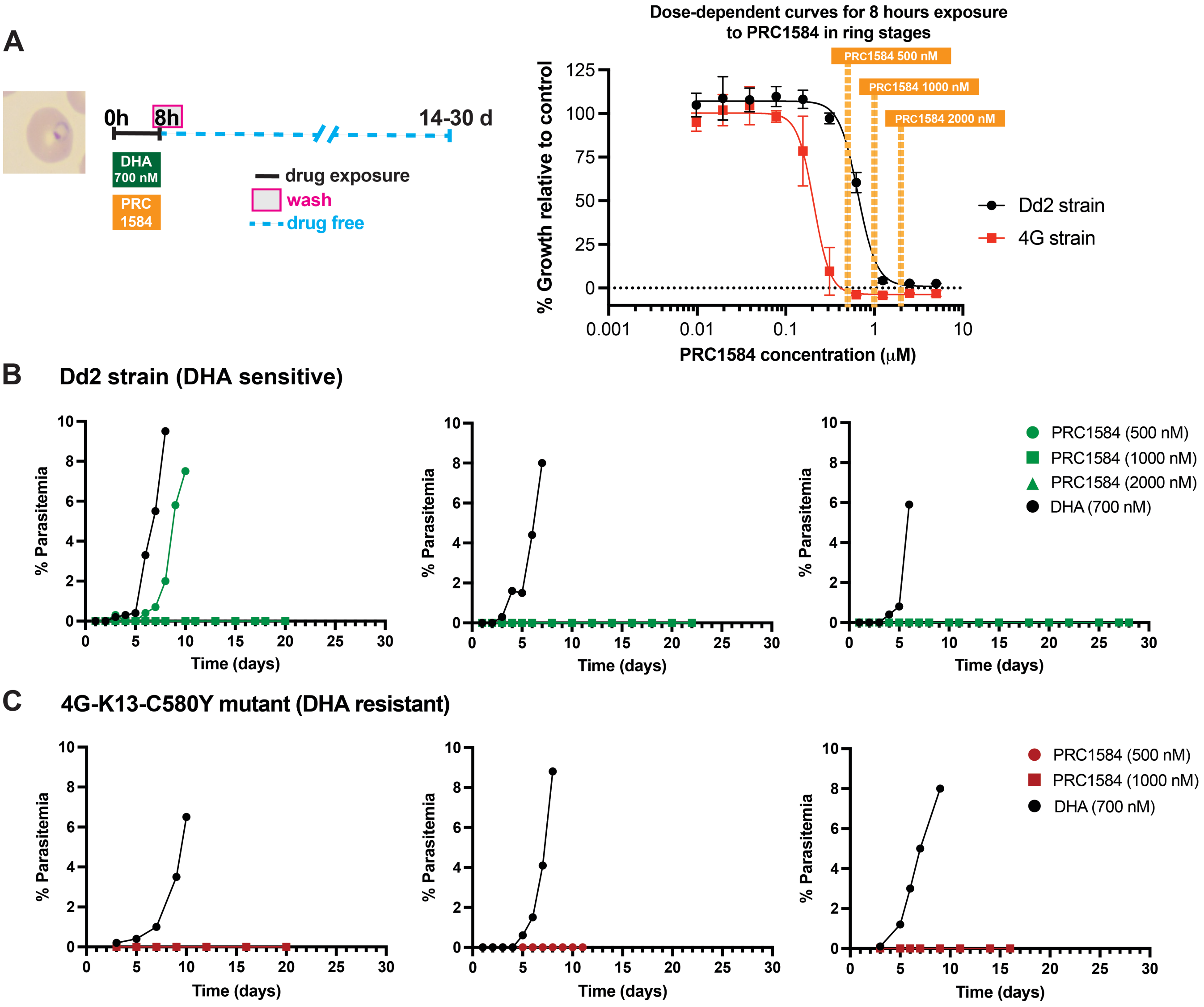

Figure S8. Ring-stage recrudescence assays with varying concentrations of PRC1584. **A)** Schematic of the experimental design used to monitor recovery of parasite after 8 hours of drug exposure at different concentrations in highly synchronous ring-stage cultures (6-8 h post-invasion). After drug removal, cultures were maintained in drug-free medium and monitored daily by Giemsa-stained thin blood smears. X-axis is shown on a logarithmic scale for the dose-dependent curves obtained after 8 hours of exposure to PRC1584. **B)** Recrudescence profiles for the Dd2 strain (K13-WT, DHA-sensitive) following treatment with PRC1584 at 500 nM (green circles), 1000 nM (green squares), or 2000 nM (green triangles) compared to DHA at 700 nM (black circles). **C)** Recrudescence profiles for the 4G-K13-C580Y mutant strain (DHA-resistant) following treatment with PRC1584 at 500 nM (red circles) or 1000 nM (red squares) compared to DHA at 700 nM (black circles). Each panel represents an independent experiment, with parasitemia plotted as % infected erythrocytes over time after treatment.

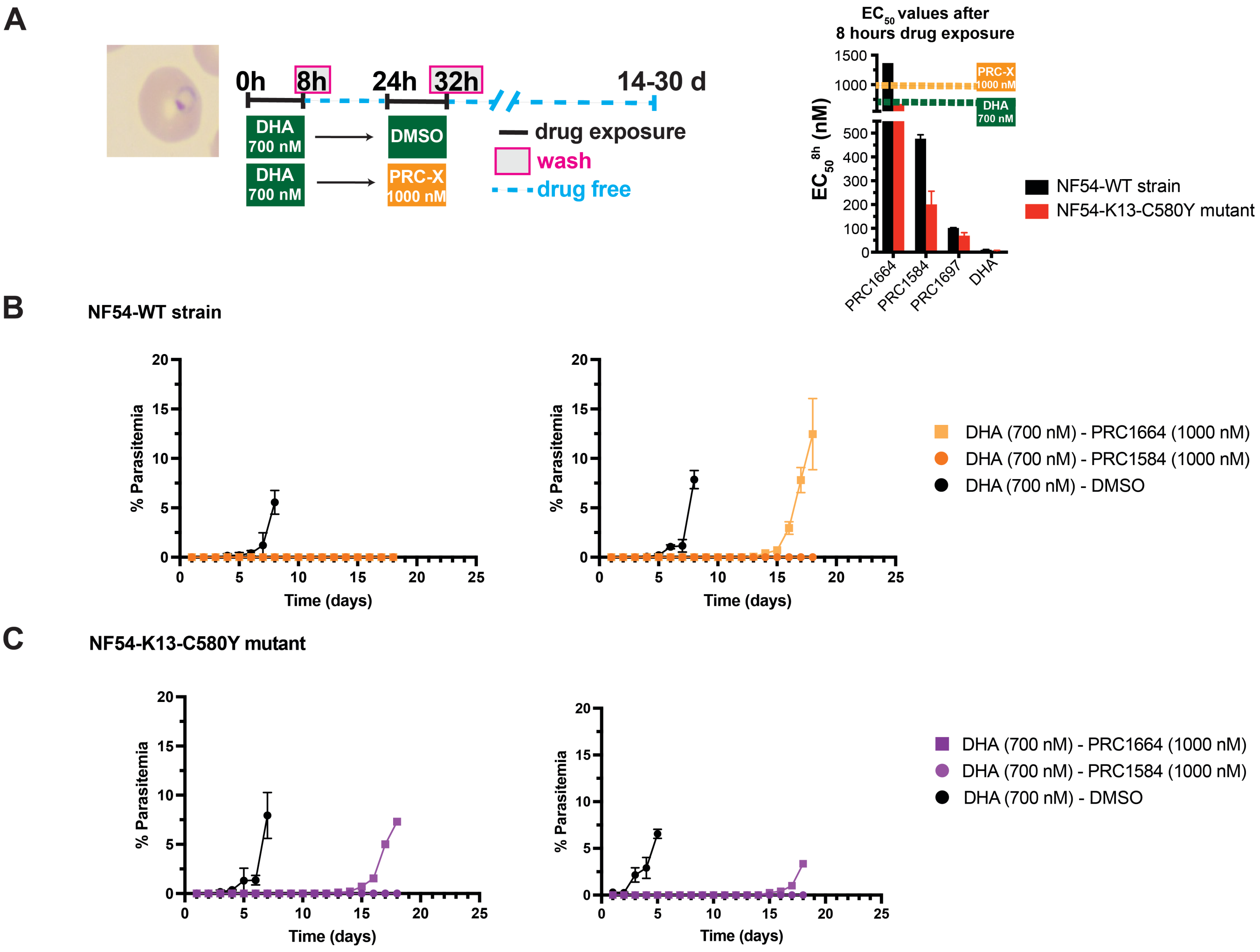

Figure S9. Effect of PRC1584 and PRC1664 on DHA-induced dormant parasites. **A)** Schematic of the experimental design used to monitor parasite recovery following 8 hours of drug exposure in DHA-induced dormant cultures. Highly synchronous ring stage parasites (6-8 h post-invasion) were first treated with 700 nM DHA for 8 hours (0–8 h), washed, and 16 hours after DHA treatment was removed, parasites were treated with 1000 nM of PRC1584 or PRC1664, or DMSO as control. Cultures were subsequently maintained in drug-free medium. Graph depicts the 8-hours EC_{50} values as shown in Figure 4B. **B)** Recrudescence profiles for the NF54-WT strain (DHA-sensitive) cultures treated with DHA followed by PRC1664 (orange squares), PRC1584 (orange circles), or DMSO (black circles). **C)** Recrudescence profiles for the NF54-K13-C580Y strain (DHA-resistant) cultures under the same treatment conditions (PRC1664, purple squares; PRC1584, purple diamonds; DMSO, black circles). Each panel shows data from a separate experiment, which was independently evaluated by two microscopists under blinded conditions.

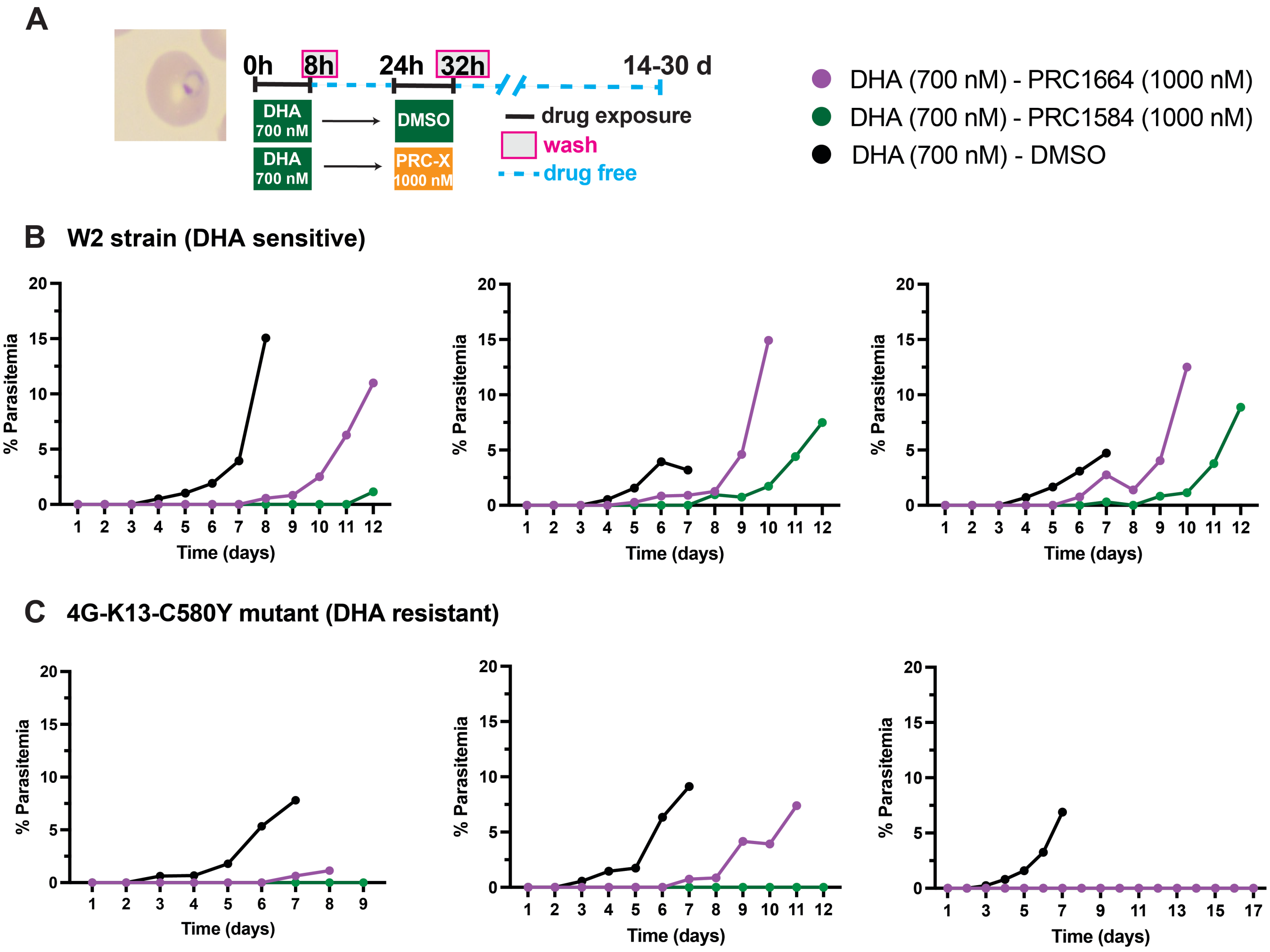

Figure S10. Initial studies evaluating the effect of PRC1584 and PRC1664 on DHA-induced dormant parasites in W2 strain (DHA-sensitive) and 4G-K13-C580Y mutant strain (DHA-resistant). **A)** Schematic of the experimental design used to monitor parasite recovery following 8 hours of drug exposure in DHA-induced dormant cultures. Highly synchronous ring stage parasites (6-8 h post-invasion) were first treated with 700 nM DHA for 8 hours (0–8 h), washed, and 16 hours after DHA treatment was removed, parasites were treated with 1000 nM of PRC1584 or PRC1664, or DMSO as control. Cultures were subsequently maintained in drug-free medium. **B)** Recrudescence profiles for the W2 strain (DHA-sensitive) after treatment with DHA followed by PRC1664 (purple), PRC1584 (green), or DMSO (black). **C)** Recrudescence profiles for the 4G-K13-C580Y mutant strain (DHA-resistant) under the same treatment conditions (PRC1664, purple; PRC1584, green; DMSO, black). Each panel represents an independent experiment, with parasitemia plotted as % infected erythrocytes over time after treatment.

Materials and Methods

Chemicals. Dihydroartemisinin was obtained from Sigma-Aldrich (St. Louis, MO). PRC1584, PRC1697 and PRC1664 were synthesized and purified, as previously described[1, 2]. All compounds were reconstituted at 10 mM in DMSO.

***Plasmodium falciparum* cultures.** The *P. falciparum* strain Dd2 (MRA-150), 3D7 (MRA-102) and NF54 (MRA-1000) were obtained from MR4 (ATCC, Manassas, VA; BEI Resources, NIAID, NIH). The *P. falciparum* 4G strain containing the PfK13-C580Y mutation was kindly supplied by Denis Kyle[3]. The *P. falciparum* NF54-PfK13-C580Y and Dd2-PfK13-R549T lines were kindly supplied by David Fidock[4]. The *P. falciparum* Pikine-Pfcoronin-R100K-E107V mutant line and the parental Pikine strain were kindly supplied by Dyann Wirth[5]. All *P.* falciparum strains were maintained in O^+ positive human erythrocytes (Grifols, Memphis, TN, USA) at 5% hematocrit in RPMI 1640 media (Thermo Fisher Scientific, Waltham, MA) containing 2 g/L glucose, 5.94 g/L HEPES, 2.3 g/L sodium bicarbonate, 5 g/L Albumax I, and 50 mg/L hypoxanthine. All reagents were obtained from Sigma-Aldrich (St. Louis, MO). Media was supplemented with 20 mg/L of gentamicin (Thermo Fisher Scientific, Waltham, MA). Parasite cultures were maintained at 37°C under reduced oxygen conditions (5% CO_2, 5% O_2, and 90% N_2) with shaking. Highly synchronous ring stage cultures (>98%) were obtained through two consecutive 5% sorbitol treatments (Sigma-Aldrich, St. Louis, MO) performed 6 hours apart.

***In vitro* selection of drug resistance.** Resistance selection for PRC1584 was assessed using both single-step and pulse methods as outlined in Figure S1[6-8]. For the single step selection, inoculums of 1 x 10^7 and 1 x 10^9 asynchronous parasites from newly cloned *P. falciparum* 3D7 strain were subjected to continuous drug pressure at twice and five times the 72 hours EC_{50} value (67 ± 5 nM), respectively. Each condition was performed in three independent cultures. For the first 7 days, media containing PRC1584 was replaced daily, then every other day until day 14. A parallel control culture was maintained under identical conditions with an equal volume of DMSO. After 14 days, drug pressure was removed, and cultures were monitored daily by Giemsa-stained smears with media changes every other day for 60 days. Fresh blood (50 µL) was added weekly to sustain culture viability.

For the pulse method (Figure S1), three independent inoculums of 1 x 10^7 asynchronous or ring stage parasites from the same 3D7 clone were briefly pulsed with eight times the EC_{50} value of PRC1584 (400 nM) for 6 hours. Cultures were washed thrice with drug-free media and transferred to a new flask. Parasites were allowed to recover to 1 x 10^7 parasites and pulsed again with 400 nM PRC1584. Cultures underwent four cycles of recovery and re-exposure; however, parasites ultimately failed to recrudesce after the fourth pulse.

***P. falciparum* morphological assessments.** Ring stage NF54-WT strain parasite cultures (>98%) were synchronized by two consecutive 5% sorbitol treatment (Sigma-Aldrich, St. Louis, MO), administered 6 hours apart, 48 hours prior to the initiation of the experiment. All cultures were maintained at 37°C under reduced oxygen conditions (5% CO_2, 5% O_2, and 90% N_2) with continuous shaking. To evaluate morphological alterations following drug treatment, compounds were applied at specified concentrations and durations detailed in each figure legend. Thin blood smears were prepared at designated time points, fixed with 100% methanol (Sigma-Aldrich, St. Louis, MO, USA), and stained for 15 minutes using a 20% Giemsa solution (Sigma-Aldrich; diluted in deionized water). Following smear preparation, parasites were immediately gassed and returned to 37°C with shaking. Morphological growth phenotypes were assessed in biological duplicates.

HepG2 and liver stage assays. HepG2 cells (*Homo sapiens* hepatoblastoma, ATCC) were maintained in collagen-coated T-75 flasks with sugar-free DMEM (Gibco), supplemented with 10% FBS (Corning), 25 mM glucose (Millipore-Sigma), 1 mM sodium pyruvate (Corning), 5 µg/mL penicillin, 5 µg/mL streptomycin, 10 µg/mL neomycin, and 2 mM L-glutamine (Gibco). Cultures were incubated at 37°C in a humidified atmosphere containing 5% CO_2. Cells were detached using TrypLE (Gibco) once cultures reached 60–90% confluence. Cell density was determined via trypan blue exclusion with a hemocytometer. Subsequently, 17,500 cells per well were dispensed into collagen-coated 384-well plates (Greiner Bio-One) using a Biomek NX^P automated workstation (Beckman Coulter), 24 hours prior to sporozoite infection.

Luciferase-expressing *P. berghei* ANKA strain GFP-Lucama1-eef1a (line 1052cl1) was obtained from the Sporocore at the University of Georgia, as previously described[9]. Sporozoites were isolated according to established protocols, utilizing bicarbonate-free RPMI (KD Medical) as the collection buffer[10]. HepG2 cells were infected with 2,000 sporozoites per well. At three hours post-infection, 40 nL of each compound dilution was transferred from dose–response source plates to assay plates using a pin tool (V&P Scientific) mounted on a Biomek NX^P, achieving a final test concentration of 1x in media. The plates were incubated for 44 hours at 37°C in a humidified atmosphere containing 5% CO_2. After incubation, plates were fixed with 4% paraformaldehyde (Thermo Scientific) in PBS for 20 minutes. Following fixation, the plates were washed twice by adding and removing 20 µL of PBS per well. Plates were stained with 50 ng/mL mouse monoclonal antibody 13.3 (anti-GAPDH) sourced from The European Malaria Reagent Repository (http://www.malariaresearch.eu) diluted in a permeabilization and blocking buffer containing 0.3% Triton X and 1% BSA, and incubated overnight at 4°C. After three PBS washes, plates were treated with 2 µg/mL goat anti-mouse AlexaFluor 488 (Invitrogen), diluted in stain buffer, and incubated overnight at 4°C. Subsequently, three PBS washes were performed before counterstaining

with 10 μg/mL Hoechst 33342 (Invitrogen) at room temperature for 30 minutes, followed by two additional washes. Imaging was conducted using an ImageXpress Micro Confocal high content system (Molecular Devices). Schizont number and area were normalized using positive (MMV390048) and negative (DMSO) controls, and hepatocyte nuclei counts were used as an indicator of toxicity.

Determination of the Minimum Inoculum for Resistance (MIR) for PRC1584 based on continuous drug pressure selection. This assay was conducted at Columbia University under a contract with Medicines for Malaria Venture. The EC_{50} and EC_{90} was first experimentally determined in duplicate in the *P. falciparum* Dd2-B2 clone. Continuous drug pressure selection was set up using 2 x 10^5 Dd2-B2 parasites in each well of a 96-well plate at a concentration of 3 x EC_{90} (408.3 nM). Parasites cleared within the first 4 days. Drug pressure was maintained at 3 x EC_{90} over 60 days, and cultures were screened three times weekly by flow cytometry and smearing. Wells were considered positive for recrudescence when the overall parasitemia reached 0.3%, and parasites were seen on a blood smear. No recrudescence was observed over the course of this selection. The study was run alongside a DSM265 (57.5 nM) control, which yielded 68 recrudescent wells out of 96 (MIR 2.8 x 10^5, Log10 MIR 5.45).

***P. falciparum* male/female gamete formation assay.** This assay was conducted at Imperial College London under a contract with Medicines for Malaria Venture. This assay assesses the viability of mature stage V gametocytes as reported by their ability to undergo onward development and form gametes[11-14]. Compounds were incubated in a dose-dependent manner with mature stage V gametocytes for 48 hours in 384 well plates before gamete formation was triggered by a drop in temperature and addition of xanthurenic acid. Twenty-five minutes post-triggering gamete formation, male gamete exflagellation was recorded and quantified by automated microscopy. The plates were then incubated at 26°C for a further 24 hours and the female gamete formation of the same samples was assessed by live staining using a fluorophore-conjugated αPfs25 antibody specific for female gametes and quantified by automated microscopy. Gamete formation was expressed as a percent inhibition, taking into consideration DMSO-negative control wells and 10 μM methylene blue-positive control wells, with methylene blue being a potent inhibitor of the functional viability of male and female stage V gametocytes as previously reported[14]. This assay identifies compounds that either directly kill the male/female gametocyte, or “sterilize” the male/female gametocyte thus preventing gamete formation or interfere with the process of gamete formation itself.

Stage-specific gametocytocidal activity. This assay was conducted at the University of Pretoria under a contract with Medicines for Malaria Venture. Gametocytes were robustly induced from the PfNF54-pfs16-GFP-luc reporter line. Drug assays were initiated on immature gametocytes on day 5-6 (QC: >30% conversion, gametocytemia >3%, viable by hydroethidine staining, >90% stage II/III distribution) or on mature gametocytes on day 13 (QC: gametocytemia ~3%, >95% mature stage V gametocytes, functional gamete formation)[15]. For immature and mature gametocytes, compounds were evaluated with 48 hours drug pressure (2% gametocytemia, 1.5% hematocrit) at 37ºC under hypoxic conditions (90% N_2, 5% O_2, and 5% CO_2). After 48 hours, luciferase activity was measured in 20 µL parasite lysates by adding 50 µL luciferin substrate (Promega Luciferase Assay System) at room temperature. Bioluminescence detection was conducted with an integration constant of 10 seconds using the GloMax® Multi Detection System, operated with Instinct® Software. Dose-response experiments include 9 points of two-fold dilution per curve, a negative control (vehicle). Methylene blue at 5 μM (immature gametocyte inhibition: 97 ± 5%; mature gametocyte inhibition: 93 ± 2%) and MMV390048 at 5 μM (immature gametocyte inhibition: 98 ± 1%; mature gametocyte inhibition: 97 ± 1%) were used as positive controls. The EC_{50} values were determined with a non-linear curve fitting normalized to maximum and minimum inhibition (DMSO control wells), using GraphPad Prism (GraphPad Software, Inc.). The assays were conducted in three biological replicates, each with technical triplicates.

References

(1) Mathew, J.; Ding, S.; Kunz, K. A.; Stacy, E. E.; Butler, J. H.; Haney, R. S.; Merino, E. F.; Butschek, G. J.; Rizopoulos, Z.; Totrov, M.; et al. Malaria Box-Inspired Discovery of N-Aminoalkyl-beta-carboline-3-carboxamides, a Novel Orally Active Class of Antimalarials. *ACS Med Chem Lett* **2022**, *13* (3), 365-370. DOI: 10.1021/acsmedchemlett.1c00663.

(2) Mathew, J.; Zhou, B.; Haney, R. S.; Kunz, K. A.; Do Amaral, L. S.; Roy Chowdhury, R.; Butler, J. H.; Li, H.; Chakraborty, A. J.; Tabassum, A.; et al. beta-Carboline-3-carboxamide Antimalarials: Structure-Activity Relationship, ADME-Tox Studies, and Resistance Profiling. *ACS Infect Dis* **2024**, *10* (11), 3951-3962. DOI: 10.1021/acsinfecdis.4c00653 From NLM Medline.

(3) Hott, A.; Casandra, D.; Sparks, K. N.; Morton, L. C.; Castanares, G. G.; Rutter, A.; Kyle, D. E. Artemisinin-resistant Plasmodium falciparum parasites exhibit altered patterns of development in infected erythrocytes. *Antimicrob Agents Chemother* **2015**, *59* (6), 3156-3167. DOI: 10.1128/aac.00197-15 From NLM.

(4) Gnadig, N. F.; Stokes, B. H.; Edwards, R. L.; Kalantarov, G. F.; Heimsch, K. C.; Kuderjavy, M.; Crane, A.; Lee, M. C. S.; Straimer, J.; Becker, K.; et al. Insights into the intracellular localization, protein associations and artemisinin resistance properties of Plasmodium falciparum K13. *PLoS Pathog* **2020**, *16* (4), e1008482. DOI: 10.1371/journal.ppat.1008482 From NLM Medline.

(5) Demas, A. R.; Sharma, A. I.; Wong, W.; Early, A. M.; Redmond, S.; Bopp, S.; Neafsey, D. E.; Volkman, S. K.; Hartl, D. L.; Wirth, D. F. Mutations in Plasmodium falciparum actin-binding protein coronin confer reduced artemisinin susceptibility. *Proc Natl Acad Sci U S A* **2018**, *115* (50), 12799-12804. DOI: 10.1073/pnas.1812317115 From NLM Medline.

(6) Bremers, E. K.; Butler, J. H.; Do Amaral, L. S.; Merino, E. F.; Almolhim, H.; Zhou, B.; Baptista, R. P.; Totrov, M.; Carlier, P. R.; Cassera, M. B. Stereospecific Resistance to N2-Acyl Tetrahydro-beta-carboline Antimalarials Is Mediated by a PfMDR1 Mutation That Confers Collateral Drug Sensitivity. *ACS Infect Dis* **2025**, *11* (2), 529-542. DOI: 10.1021/acsinfecdis.4c01001 From NLM Medline.

(7) Butler, J. H.; Baptista, R. P.; Valenciano, A. L.; Zhou, B.; Kissinger, J. C.; Tumwebaze, P. K.; Rosenthal, P. J.; Cooper, R. A.; Yue, J. M.; Cassera, M. B. Resistance to Some But Not Other Dimeric Lindenane Sesquiterpenoid Esters Is Mediated by Mutations in a Plasmodium falciparum Esterase. *ACS Infect Dis* **2020**, *6* (11), 2994-3003. DOI: 10.1021/acsinfecdis.0c00487 From NLM Medline.

(8) Ng, C. L.; Fidock, D. A. Plasmodium falciparum In Vitro Drug Resistance Selections and Gene Editing. *Methods Mol Biol* **2019**, *2013*, 123-140. DOI: 10.1007/978-1-4939-9550-9_9 From NLM Medline.

(9) Maher, S. P.; Vantaux, A.; Cooper, C. A.; Chasen, N. M.; Cheng, W. T.; Joyner, C. J.; Manetsch, R.; Witkowski, B.; Kyle, D. A Phenotypic Screen for the Liver Stages of Plasmodium vivax. *Bio Protoc* **2021**, *11* (23), e4253. DOI: 10.21769/BioProtoc.4253 From NLM PubMed-not-MEDLINE.

(10) Nguyen, G. B.; Cooper, C. A.; McWhorter, O.; Sharma, R.; Elliot, A.; Ruberto, A.; Freitas, R.; Pathak, A. K.; Kyle, D. E.; Maher, S. P. Screening the Global Health Priority Box against Plasmodium berghei liver stage parasites using an inexpensive luciferase detection protocol. *Malar J* **2024**, *23* (1), 357. DOI: 10.1186/s12936-024-05155-y From NLM Medline.

(11) Delves, M. J.; Miguel-Blanco, C.; Matthews, H.; Molina, I.; Ruecker, A.; Yahiya, S.; Straschil, U.; Abraham, M.; Leon, M. L.; Fischer, O. J.; et al. A high throughput screen for next-generation leads targeting malaria parasite transmission. *Nat Commun* **2018**, *9* (1), 3805. DOI: 10.1038/s41467-018-05777-2 From NLM Medline.

(12) Delves, M. J.; Ruecker, A.; Straschil, U.; Lelievre, J.; Marques, S.; Lopez-Barragan, M. J.; Herreros, E.; Sinden, R. E. Male and female Plasmodium falciparum mature gametocytes show different responses

to antimalarial drugs. *Antimicrob Agents Chemother* **2013**, *57* (7), 3268-3274. DOI: 10.1128/AAC.00325-13 From NLM Medline.

(13) Delves, M. J.; Straschil, U.; Ruecker, A.; Miguel-Blanco, C.; Marques, S.; Dufour, A. C.; Baum, J.; Sinden, R. E. Routine in vitro culture of P. falciparum gametocytes to evaluate novel transmission-blocking interventions. *Nat Protoc* **2016**, *11* (9), 1668-1680. DOI: 10.1038/nprot.2016.096 From NLM Medline.

(14) Ruecker, A.; Mathias, D. K.; Straschil, U.; Churcher, T. S.; Dinglasan, R. R.; Leroy, D.; Sinden, R. E.; Delves, M. J. A male and female gametocyte functional viability assay to identify biologically relevant malaria transmission-blocking drugs. *Antimicrob Agents Chemother* **2014**, *58* (12), 7292-7302. DOI: 10.1128/AAC.03666-14 From NLM Medline.

(15) Reader, J.; van der Watt, M. E.; Birkholtz, L. M. Streamlined and Robust Stage-Specific Profiling of Gametocytocidal Compounds Against Plasmodium falciparum. *Front Cell Infect Microbiol* **2022**, *12*, 926460. DOI: 10.3389/fcimb.2022.926460 From NLM Medline.
